# Construction of Unified Human Antimicrobial and Immunomodulatory Peptide Database and Examination of Antimicrobial and Immunomodulatory Peptides in Alzheimer’s Disease Using Network Analysis of Proteomics Datasets

**DOI:** 10.3389/fgene.2021.633050

**Published:** 2021-04-28

**Authors:** Ajneesh Kumar, Vo Minh Doan, Balázs Kunkli, Éva Csősz

**Affiliations:** ^1^Proteomics Core Facility, Department of Biochemistry and Molecular Biology, Faculty of Medicine, University of Debrecen, Debrecen, Hungary; ^2^Biomarker Research Group, Department of Biochemistry and Molecular Biology, Faculty of Medicine, University of Debrecen, Debrecen, Hungary; ^3^Doctoral School of Molecular Cell and Immune Biology, Faculty of Medicine, University of Debrecen, Debrecen, Hungary

**Keywords:** antimicrobial immunomodulatory peptides, UDAMP database, Alzheimer’s disease, APP, network analysis

## Abstract

The reanalysis of genomics and proteomics datasets by bioinformatics approaches is an appealing way to examine large amounts of reliable data. This can be especially true in cases such as Alzheimer’s disease, where the access to biological samples, along with well-defined patient information can be challenging. Considering the inflammatory part of Alzheimer’s disease, our aim was to examine the presence of antimicrobial and immunomodulatory peptides in human proteomic datasets deposited in the publicly available proteomics database ProteomeXchange (http://www.proteomexchange.org/). First, a unified, comprehensive human antimicrobial and immunomodulatory peptide database, containing all known human antimicrobial and immunomodulatory peptides was constructed and used along with the datasets containing high-quality proteomics data originating from the examination of Alzheimer’s disease and control groups. A throughout network analysis was carried out, and the enriched GO functions were examined. Less than 1% of all identified proteins in the brain were antimicrobial and immunomodulatory peptides, but the alterations characteristic of Alzheimer’s disease could be recapitulated with their analysis. Our data emphasize the key role of the innate immune system and blood clotting in the development of Alzheimer’s disease. The central role of antimicrobial and immunomodulatory peptides suggests their utilization as potential targets for mechanistic studies and future therapies.

## Introduction

Antimicrobial and immunomodulatory peptides (AMPs) are produced by most of the organisms including animals and a variety of other species like bacteria, fungi, etc. (Epand and Vogel, 1999; Zhe and Guangshun, 2004). Human AMPs are elements of the innate immune system (Prashant et al., 2018) and constitute a first line of defense protecting the host from invading pathogens (Epand and Vogel, 1999; Zhe and Guangshun, 2004). They can be gene-coded peptides or small proteins, which are either constitutively expressed such as LL-37 cathelicidin, human beta defensin 1 (HBD1), dermcidin (DCD) (Bardan et al., 2004; Brandwein et al., 2017) or induced like HBD2, HBD3, and RNAse7 (Bardan et al., 2004; Brandwein et al., 2017) to fight off pathogens (Prashant et al., 2018; Jhong et al., 2019). Another group of AMPs are the ones that are cleaved off from proteins having antimicrobial activity such as lysozyme (Ibrahim et al., 2011) and S100A12 (Guangshun, 2014), or proteins with other biological function like the metabolic enzyme glyceraldehyde-3 phosphate dehydrogenase (Wagener et al., 2013), hornerin (Fleming et al., 2012; Gerstel et al., 2018), or epidermal keratin (Tam et al., 2012). It was observed that tissues that come in contact with potential pathogens may release AMPs (Ganz, 2003; Dürr et al., 2006), which, in this way, can be found in all body fluids such as sweat, saliva, tears, etc., coming in contact with the outer environment containing potential pathogens (Ganz, 2003; Kamysz et al., 2003; Prashant et al., 2018).

AMPs either interact with pathogens leading to their elimination or modulate the immune system (Prashant et al., 2018). Beside these functions, AMPs have a role in apoptosis, phagocytosis, wound healing, fertilization, etc. (Jenssen et al., 2006; Prashant et al., 2018).

From a structural point of view, AMPs are diverse biomolecules. Typically, they have a length of approximately 50 amino acids, but the range is relatively wide, with the shortest AMP with 10 amino acids (neurokinin A) to the proteins with approximately 150 amino acids such as lysozyme, RegIIIa, or RNAse 2, 3, or 7 (Guangshun, 2014). Usually AMPs have characteristic structural domains; for example, defensins have the so-called defensin fold, and cathelicidins have the highly conserved cathelicidin domain providing high stability even in harsh environmental conditions such as high salt concentration observed in body fluids (Torres and Kuchel, 2004; Wiesner and Vilcinskas, 2010; Mojsoska and Jenssen, 2015). Besides the well-defined conserved domains often found in AMPs, cationic intrinsically disordered peptides with antimicrobial activity were also identified (Latendorf et al., 2019).

Extensive studies demonstrate the role of inflammation in the pathophysiology of neurodegenerative diseases, such as Alzheimer’s disease (AD) characterized by dementia (Mendonça et al., 2019). Major pathological features of AD are the formation of senile plaques and neurofibrillary tangles in the brain, made up by the accumulation of amyloid beta (Aβ) and hyperphosphorylated tau protein, respectively (Tuppo and Arias, 2005). A correlation between the number of Aβ plaques and/or of neurofibrillary tangles and the extent of cognitive impairment was observed indicating the prominent role of these proteins in the pathophysiology of AD (Mendonça et al., 2019).

In the recent years, technologies like proteomics and genomics have played an important role in the identification of gene- or protein-level changes characteristic of AD and of candidate biomarkers for the early detection of the disease (McKetney et al., 2019; Yao et al., 2019). Extensive studies regarding postmortem brain tissue (Salobrar-Garcia et al., 2019), cerebrospinal fluid (Sathe et al., 2019; Sjödin et al., 2019), or blood (Shen et al., 2017; Dey et al., 2019) have been carried out to understand the molecular events leading to AD and to identify biomarkers that can help the differential diagnosis and the early detection of the disease. Beside Aβ and tau protein, other potential biomarkers with a role in AD were identified in various studies. Presenillin (PSEN1, PSEN2), β-site APP-cleaving enzyme 1 (BACE1), alpha-1-antitrypsin (SERPINA1), apolipoprotein E (APOE), neurosecretory VGF (VGF), and complement components were identified as biomarkers for AD (Wattamwar and Mathuranath, 2010; Sathe et al., 2019).

It was shown that the activation of microglia by the Aβ peptide results in increased secretion of pro-inflammatory cytokines. The interaction of the astrocytes with Aβ deposits can also lead to secretion of pro-inflammatory molecules like interleukins, prostaglandins, and leukotrienes, leading, in this way, to an inflammatory response (Tuppo and Arias, 2005). Based on scientific evidence, APP and its derivative Aβ have a central role in the development of AD; the Aβ oligomers can either directly injure the neurons or through the activation of microglia and astrocytes leading to progressive neuron loss and AD. In spite of the extensive data regarding the presence of Aβ and Aβ deposits, the so-called amyloid hypothesis of AD is challenged (Hardy and Selokoe, 2002). According to the current evidences, Aβ seems to have protective functions: has antimicrobial and tumor suppressor activity, promotes the recovery from brain injury, helps the maintenance of the blood–brain barrier, and regulates synaptic functions (Brothers et al., 2018). In light of these data, it is not surprising that the clinical trials targeting the accumulation of Aβ and the formation of Aβ deposits did not improve the clinical outcome of the patients, and in many cases, they led to the worsening of the symptoms (Brothers et al., 2018).

Aβ was shown to be an AMP with as potent activity as LL-37 cathelicidin (Soscia et al., 2010; Gosztyla et al., 2018) acting against various bacteria, fungi, and viruses supporting the “Pathogen Hypothesis” of AD according to which cerebral infections cause the AD (Robinson et al., 2004). Considering that none of the drugs targeting the deposition of Aβ was effective in clinical trials, the identification of Aβ and APP as AMPs led to the emergence of the “Antimicrobial Protection Hypothesis.” According to this hypothesis, Aβ is part of a defense mechanism against infection, and the chronic activation of innate immune system along with Aβ and tau pathology leads to neurodegeneration and dementia (Moir et al., 2018).

As far as AMPs have a role in innate immune response, which is involved in the pathophysiology of AD, and some AMPs were directly linked to AD, our aim was to examine in which extent the AMPs might be implicated in the pathogenesis of this neurodegenerative disease. We were eager to get information on the AMPs characteristic of the brain, cerebrospinal fluid, and blood in conjunction with AD and aimed to observe how AD perturbs the network of AMPs. At the same time, we intended to increase data reutilization; thus, for our analyses, high-quality proteomics data deposited in publicly accessible databases were used.

## Materials and Methods

### Generation of Comprehensive Human Antimicrobial and Immunomodulatory Peptide Database (UDAMP Database)

All human AMPs listed in the Collection of Anti-Microbial Peptides (CAMP) (Thomas et al., 2009), Antimicrobial Peptide Database (APD) (Zhe and Guangshun, 2004), Database of Antimicrobial Peptides (dbAMP) (Jhong et al., 2019), Linking Antimicrobial Peptides (LAMP) (Zhao et al., 2013), and Database of Antimicrobial Activity and Structure of Peptides (DBAASP) (Gogoladze et al., 2014) online databases were retrieved. PubMed (release May 15, 2020) was searched between May 2020 and June 2020 using “human antimicrobial peptide” keywords, and the hits not present in the online AMP databases were considered. Retrieved data were curated, redundancies were removed, and a unified comprehensive human antimicrobial and immunomodulatory database named University of Debrecen Antimicrobial and Immunomodulatory Peptide (UDAMP) Database was generated. Starting with the list of UniProt IDs of AMPs, the NCBI gene identifiers (Gene IDs) were obtained from UniProt (Sayers et al., 2020)^1^ through its Applications Programming Interface (API). NCBI provides cross annotations, links between gene IDs and PubMed IDs. Once given the Gene IDs, a list of PubMed IDs specifically related to these proteins were extracted from the annotation file called ‘‘gene2pubmed,’’ available on NCBI’s FTP server^2^. Based on the PubMed IDs, the article metadata was fetched via E-utilities (Sayers et al., 2020), a programming interface for the NCBI Entrez databases. Finally, we ran an offline keyword search on the article metadata set with an in-house written script.

The high human AMP sequence redundancy in the databases made the verification of individual peptide/protein sequences necessary. They were aligned against the UniProtKB/Swiss-Prot proteome database using BLASTP^3^ with the following settings: the target organism was “*Homo sapiens*,” E-threshold was set to 1e-10; scoring parameters: matrix: BLOSUM62 (default), filtering: none, gap costs: existence 11, extension 1 (default), limit of hits: 250. For each AMP, the following data fields were retrieved and integrated into the UDAMP database: protein name, gene name, UniProt ID, GI number, peptide sequence, length of peptide, antiviral, antibacterial or antifungal activity, Gram type in case of those having antibacterial activity, method of validation, database ID from where the AMP was downloaded, and the Protein Data Bank (PDB) ID indicating the structure of the AMP, when available. The source online AMP databases provided different identifiers for the listed AMPs, and to retrieve or verify the protein name, gene name, UniProt ID and GI number; the UniProt^4^ and NCBI GenBank^5^ databases were queried. The Protein Databank ID was retrieved from the RCSB Protein Databank^6^; all the other data were from the source online AMP database or scientific article deposited in PubMed.

### Examination of AMPs in the Alzheimer’s Disease Datasets

Alzheimer’s disease datasets involving human donors were retrieved primarily from the ProteomeXchange (see footnote) repository (Vizcaíno et al., 2014). All datasets from human experiments deposited before the end of 2019 were selected, where samples from patients with AD and matched non-AD controls were examined, and a comparison of AD–healthy control was carried out. The datasets involving only the examination of samples from patients with AD were omitted from our analysis. The retrieved datasets originated from the analysis of the brain, cerebrospinal fluid (CSF), or blood (Table 1).

**TABLE 1:** List of datasets used in the study.

**Dataset**	**Source**	**Tissue**	**References**
**Identifier**	**database**	**type**	
PXD009199	ProteomXchange	Brain	Measho et al., 2018
PXD008807	ProteomXchange	Brain	Xu et al., 2019
PXD012203	ProteomXchange	Brain	Adav et al., 2019
PXD004510	ProteomXchange	Brain	Wang et al., 2017
PXD005321	ProteomXchange	Brain	Hales et al., 2016
PXD000067	ProteomXchange	Brain	Bai et al., 2013
PXD014376	ProteomXchange	Brain	Higginbotham et al., 2019
PXD006122	ProteomXchange	Brain	Bereczki et al., 2018
PXD0014557	ProteomXchange	Brain	Haytural et al., 2020
PMID:30137212	PubMed	Brain	Petyuk et al., 2018
PXD007694	ProteomXchange	Brain	Cherry et al., 2018
PXD012851	ProteomXchange	CSF	Sjödin et al., 2019
PXD008098	ProteomXchange	CSF	Sathe et al., 2019
PXD0011482	ProteomXchange	Blood	Dey et al., 2019
PMID:27911324	PubMed	Blood	Shen et al., 2017

*The dataset identifier, the name of the source database, and the type of sample used for achieving the data along with references are listed for each processed dataset. CSF, cerebrospinal fluid.*

We checked which AMPs listed in the UDAMP database overlapped with the downloaded datasets and evaluated the changes in their levels. Our evaluation relied on the results of the statistical analysis performed by the authors. The AMPs whose level showed a statistically significant change in AD samples compared with controls were assigned with labels “Increase” or “Decrease,” while the AMPs present without any significant statistical change were labeled “No significant change.” The AMPs that were not present in the examined dataset were given a value of “0.”

### Network Analysis

Network analysis was performed on AMPs present in the UDAMP Database and AMPs identified in the retrieved datasets. For network analysis and visualization, we used Cytoscape (Shannon et al., 2003) with the STRING-DB v11^7^ (Szklarczyk et al., 2019) and ClueGO v2.5.7 + CluePedia v1.5.7 (Bindea et al., 2009, 2013) plugins. Query proteins were uploaded to the STRING-DB, where networks were generated with the interaction score set to 0.9 confidence limit. In case of networks containing the AMPs with statistically significant change in AD, the 50 first shells of interactors were selected. The generated interaction networks were exported into the Cytoscape and examined using the “Analyze Network” option available in the “Tools” menu of Cytoscape. Pathway analysis was performed by the ClueGO (Bindea et al., 2009) using default settings, and the number of gene visualization threshold was set to 1,000 in CluePedia. The betweenness centrality, the degree of distribution, and number of undirected and directed nodes of the network were obtained. The Cytoscape network was imported into the ClueGO v2.5.7 + CluePedia v1.5.7 plugin. The gene function analysis was performed for the Cytoscape network genes by CluePedia v1.5.7 using default settings. The parameters used for the gene interaction network analysis was the activation, binding, co-expression, and inhibition selected form the STRING-DB database. Pathway analysis was performed by the DAVID (Huang et al., 2009). For identifier, the official gene symbol was set, “*Homo sapiens*” was the chosen species, and list type was chosen for gene list. A new list file was created in the DAVID, which was further submitted for gene ontology (GO) analysis. Based on the gene counts, the top 10 enriched pathways were plotted.

## Results

### Generation of the Comprehensive Human Antimicrobial and Immunomodulatory Peptide Database (UDAMP Database)

Our aim was to examine the AMPs and AMP interaction networks characteristic for AD. In order to achieve our goals, first, a unified and comprehensive database for the human AMPs was generated named University of Debrecen Antimicrobial and Immunomodulatory Peptide (UDAMP) Database. For the generation of UDAMP Database, data obtained from public repositories was used. All the non-redundant human AMPs available till the end of 2019 have been collected and deposited into the UDAMP Database currently containing 186 human AMPs. In the case of each AMP, the protein and gene identifiers [gene and protein name, UniProt ID, GI number, the Protein Databank ID (PDB ID)] and the database from which it was retrieved, the peptide sequence, and the type of antimicrobial activity were listed. In the case of AMPs having antibacterial activity, the Gram type of bacteria, which they influence, was indicated as well. To the “Validation” cell data regarding the type of validation of antimicrobial activity (experimentally validated or predicted) was introduced; Table 2^8^).

**TABLE 2:** Antimicrobial and immunomodulatory proteins listed in the comprehensive human antimicrobial immunomodulatory peptide (UDAMP) database.

**Gene name**	**UniProt ID**	**Protein name**
FAU	P62861	40S ribosomal protein S30
ADM	P35318	Adrenomedullin
SERPINA1	P01009	Alpha-1-antitrypsin
SNCA	P37840	Alpha-synuclein
APP	P05067	Amyloid-beta precursor protein
ANG	P03950	Angiogenin
SLPI	P03973	Antileukoproteinase
ATP5F1B	P06576	ATP synthase subunit beta, mitochondrial
AZU1	P20160	Azurocidin
BPI	P17213	Bactericidal permeability-increasing protein
B2M	P61769	Beta-2-microglobulin
CSN2	P05814	Beta-casein
DEFB105B	B2RU30	Beta-defensin
PRG2	P13727	Bone marrow proteoglycan
BST2	Q10589	Bone marrow stromal antigen 2
BPIFA1	Q9NP55	BPI fold-containing family A member 1 and 2
BPIFB3	P59826	BPI fold-containing family B member 3 and 4
CALCA	P06881	Calcitonin gene-related peptide 1
CAMP	P49913	Cathelicidin antimicrobial peptide
CTSG	P08311	Cathepsin G
CCL1	P22362	C-C motif chemokine 1, 4, 8, 13, 17, 18, 19, 20, 21, 22, 24, 25, 27, 28
CHGA	P10645	Chromogranin-A
CLU	P10909	Clusterin
CXCL2	P19875	C-X-C motif chemokine 2, 3, 6, 9, 10, 11, 13, 14
CST11	Q9H112	Cystatin-11
CST5	P28325	Cystatin-D
CST4	P01036	Cystatin-S
CST2	P09228	Cystatin-SA
CST1	P01037	Cystatin-SN
DEFA3	Q6EZE9	Defensin, alpha 3, neutrophil-specific
DEFA5	Q01523	Defensin-5 and 6
DMBT1	Q9UGM3	Deleted in malignant brain tumors 1 protein
DCD	P81605	Dermcidin
APOBEC3G	Q9HC16	DNA dC-> dU-editing enzyme APOBEC-3G
PI3	P19957	Elafin
RNASE3	P12724	Eosinophil cationic protein
CCL11	P51671	Eotaxin
LACRT	Q9GZZ8	Extracellular glycoprotein lacritin
FGA	P02671	Fibrinogen alpha chain
FLG2	Q5D862	Filaggrin-2
FURIN	P09958	Furin
GALP	Q9UBC7-2	Galanin-like peptide
LGALS3	P17931	Galectin-3
GAPDH	P04406	Glyceraldehyde-3-phosphate dehydrogenase
GNLY	P22749	Granulysin
CXCL1	P09341	Growth-regulated alpha protein
GBP1	P32455	Guanylate-binding protein 1 and 2
HP	P00738	Haptoglobin beta chain
SERPIND1	P05546	Heparin cofactor 2
HAMP	P81172	Hepcidin
HTN1	P15515	Histatin-1, 3, 5, and 8
HRG	P04196	Histidine-rich glycoprotein
H2AFJ	Q9BTM1	Histone H2A.J
HIST1H2BC	P62807	Histone H2B type 1-C/E/F/G/I
HRNR	Q86YZ3	Hornerin
IFNA2	P01563	Interferon alpha-2
IFNL3	Q8IZI9	Interferon lambda-3
MX1	P20591	Interferon-induced GTP-binding protein Mx1
IFIH1	Q9BYX4	Interferon-induced helicase C domain-containing protein 1
ISG20	Q96AZ6	Interferon-stimulated gene 20 kDa protein
IL26	Q9NPH9	Interleukin-26
CXCL8	P10145	Interleukin-8
IAPP	P10997	Islet amyloid polypeptide
KLK5	Q9Y337	Kallikrein-5
KRT6C	P48668	Keratin, type II cytoskeletal 6C
KNG1	P01042	Kininogen-1
LTF	P02788	Lactotransferrin
LCN1	P31025	Lipocalin-1
LEAP2	Q969E1	Liver-expressed antimicrobial peptide 2
XCL1	P47992	Lymphotactin
LYZ	P61626	Lysozyme C
SCGB2A1	O75556	Mammaglobin-B
POMC	P01189	Melanocyte-stimulating hormone alpha(Pro-opiomelanocortin)
MUC7	Q8TAX7	Mucin-7
NPPB	P16860	Natriuretic peptides B
NPY	P01303	Neuropeptide Y
VGF	O15240	Neurosecretory protein VGF
NTS	P30990	Neurotensin
DEFA1	P59665	Neutrophil defensin 1, 3 and 4
LCN2	P80188	Neutrophil gelatinase-associated lipocalin
HMGN2	P05204	Non-histone chromosomal protein HMG-17
PYY	P10082	Peptide YY
PGLYRP3	Q96LB9	Peptidoglycan recognition protein 3 and 4
PRF1	P14222	Perforin-1
PLA2G2A	P14555	Phospholipase A2, membrane associated
PPBP	P02775	Platelet basic protein
PF4	P02776	Platelet factor 4
PIGR	P01833	Polymeric immunoglobulin receptor
PRR4	Q16378	Proline-rich protein 4
TOR2A	Q8N2E6	Prosalusin
TAC1	P20366	Protachykinin-1
GPR15L	Q6UWK7	Protein GPR15L
S100A7	P31151	Protein S100-A7
S100A8	P05109	Protein S100-A8
S100A9	P06702	Protein S100-A9
S100A12	P80511	Protein S100-A12
F2	P00734	Prothrombin
IQGAP2	Q13576	Ras GTPase-activating-like protein IQGAP2
ROMO1	P60602	Reactive oxygen species modulator 1
REG3A	Q06141	Regenerating islet-derived protein 3-alpha
RETN	Q9HD89	Resistin
RARRES2	Q99969	Retinoic acid receptor responder protein 2
DLC1	Q96QB1	Rho GTPase-activating protein 7
RNASE8	Q8TDE3	Ribonuclease K6, 7 and 8
SALV	Q86YR0	Salivary gland antimicrobial salvic
SCGB1D1	O95968	Secretoglobin family 1D member 1
SEMG1	P04279	Semenogelin-1 and 2
SPINK9	Q5DT21	Serine protease inhibitor Kazal-type 9
SPAG11B	Q08648	Sperm-associated antigen 11B
CXCL12	P48061	Stromal cell-derived factor 1
DCD	P81605	Survival-promoting peptide, Y-P30
TSLP	Q969D9	Thymic stromal lymphopoietin
SP1	P08047	Transcription factor Sp1
VIP	P01282	VIP peptides
WFDC12	Q8WWY7	WAP four-disulfide core domain protein12
AZGP1	P25311	Zinc-alpha-2-glycoprotein
ZG16B	Q96DA0	Zymogen granule protein 16 homolog B

*For each antimicrobial and immunomodulatory peptide (AMP), the gene name, UnirPot ID, and protein name is listed. In case of proteins with more isoforms, the gene name and UniProt ID of the first entry was kept, and isoforms are indicated in the protein name. More details regarding the proteins, along with the peptide sequences, can be found at https://doi.org/10.6084/m9.figshare.13590083.v2.*

**FIGURE 1:**
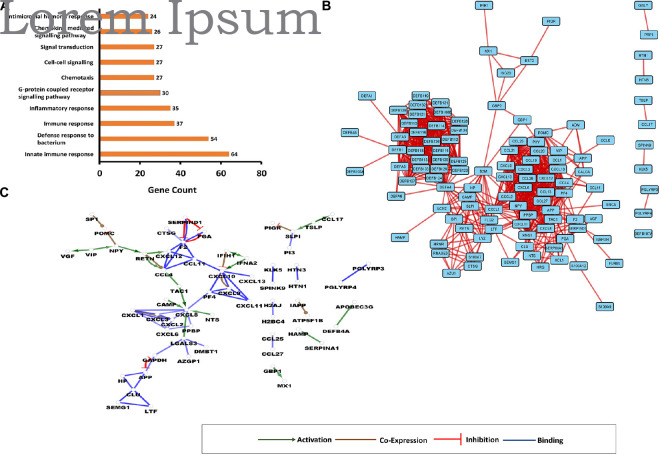
Interaction networks involving human antimicrobial and immunomodulatory peptides (AMPs). **(A)** Top 10 enriched gene ontology (GO) terms for AMPs present in comprehensive human antimicrobial and immunomodulatory peptide (UDAMP) Database. The enriched GO terms are shown on the “*y*” axis, while the gene counts are shown on the “*x*” axis. **(B)** Protein–protein interaction (PPI) network. Each rectangle represents a protein, and the line represents the interactions. **(C)** Gene interaction network (GIN). Circles represent a gene/protein, and the lines indicate interactions. The lines with arrow represent activation, blocking lines represent inhibition, and simple lines represent protein–protein interaction. Line color indicates the type of interaction: green color refers to activation, red color to inhibition, blue color binding, whereas brown color shows co-expression. On all panels, the proteins are labeled with their gene name. The higher resolution images of the networks are presented in Supplementary Figure 1.

In order to get information on the protein–protein interaction network of AMPs, STRING-DB and Cytoscape along with the examination of GO functions were used. GO terms were examined using DAVID, the protein–protein interaction data by Cytoscape v3.8.1, whereas the gene interaction data by the CluePedia v.1.5.7. (Figure 1 and Supplementary Figure 1).

The enriched GO terms were mostly related to the regulatory processes such as cell–cell signaling, signal transduction, and G-protein-coupled receptor signaling pathway, to the antimicrobial response and the activation of immune system (Figure 1A). According to the betweenness centrality and the degree of distribution, some proteins such as APP, DEFA4, CXCL1, CXCL10, KNG1, PPBP, POMC, and PF4 were shown to play central roles in the network. Defensins formed a highly interconnected cluster having interactions with other AMPs such as SLPI, BPI, LCN2, and CAMP. The bridging molecule between the cluster of defensins and other AMPs was the hub protein DEFA4 (Figure 1B).

Considering the gene interaction networks, several regulatory subnetworks have been observed: VIP-activated VGF and NPY; this later inhibited PGMC and activated RETN, which activated CXCL12 and CCL4. CCL4 was the downstream target not only for RETN but also for TAC1, and the CXCL8 was the target for several other upstream activators: TAC1, CAMP, NTS, and LGALS3 (Figure 1C). A reciprocal inhibition was observed between APP and GAPDH, and an inhibitory loop where F2 inhibits PGA, and SERPIND1 inhibits F2 and PGA, was identified. It was observed that TSLP can activate CCL17 and SLPI, which in turn inhibits PIGR. The mutual activation of IFIH1 and IFNA2 could be observed, and IFIH1 inhibited CXCL10, while the IFNA2 activated it. Besides the networks of modulators, some solitaire activations could also be observed: SERPINA1 activated HAMP, DEFB4A activated APOBEC3G, and GBP1 activated MX1 (Figure 1C).

### Examinations of the Network of Antimicrobial and Immunomodulatory Peptides in the Brain, Cerebrospinal Fluid, and Blood

In order to examine the AMPs characteristic of AD in the brain, CSF, and blood, the high-quality proteomics data retrieved from the ProteomeXchange repository and the supplementary materials provided along with the examined data by the different research groups were used.

**FIGURE 2:**
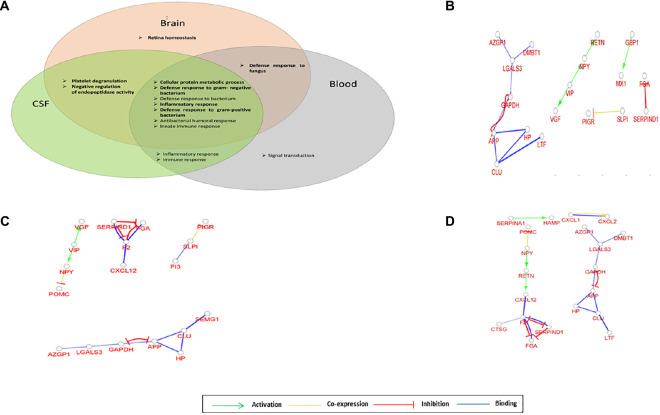
Network of AMPs characteristic for brain, cerebrospinal fluid (CSF) and blood. **(A)** Venn diagram of the top 10 enriched GO terms for AMPs identified in the brain, CSF, or blood. **(B)** Gene interaction network characteristic for AMPs identified in brain. **(C)** Gene interaction network characteristic for AMPs identified in CSF. **(D)** Gene interaction network characteristic for AMPs identified in blood. The circles represent a gene/protein and the lines indicate interactions. The lines with arrow represent activation, blocking lines represent inhibition, simple lines represent protein–protein interaction. Line color indicates the type of interaction: green color refers to activation, red color to inhibition, blue color to binding, whereas yellow color shows co-expression. On all panels the proteins are labeled with their gene name.

We processed 11 datasets providing data from the brain tissue, two datasets from the CSF, and two from the blood (Supplementary Table 1). Typically 0.5% of identified proteins belonged to the AMP family in the brain, and 1–2% in the CSF and blood. The interaction network analysis was performed, and the network of AMPs observed in the brain, CSF, and blood, respectively, was investigated (Figure 2).

The GO functions enriched in the brain, CSF, and blood were mainly related to defense against bacteria, inflammatory, and innate immune response; however, some sample-characteristic functions were observed to be enriched. The cellular protein metabolic process was more prominent in all three sample types, while the retina homeostasis was characteristic of the brain. The platelet degranulation and negative regulation of endopeptidase activity appeared in the case of the brain and CSF, and the defense response to fungi was enriched both in the brain and blood (Figure 2A and Supplementary Figure 2). In addition to the GO functions found as enriched with DAVID, GO functions related to lipid export, catecholamine secretion, viral genome replication, and hydrogen peroxide catabolism were shown to be characteristic of the brain using ClueGO. The amyloid fibril formation was characteristic of both brain and CSF, the platelet degranulation of the CSF and blood, while the regulation of tube diameter and hormone activity was shown to be enriched in CSF with ClueGO (Supplementary Figure 2). The gene interaction network analysis indicated the presence of regulatory circuits noted among AMPs originating from different parts of the body. The VIP—VGF—NPY—RETN axis in the general AMP network could be observed in the case of the brain, CSF, and blood as well, but not all components were present (Figures 2B–D). However, the inhibitory loop and the reciprocal inhibition between APP and GAPDH were present in all cases.

### Network Analysis of the Antimicrobial and Immunomodulatory Peptides Characteristic of Alzheimer’s Disease

**FIGURE 3:**
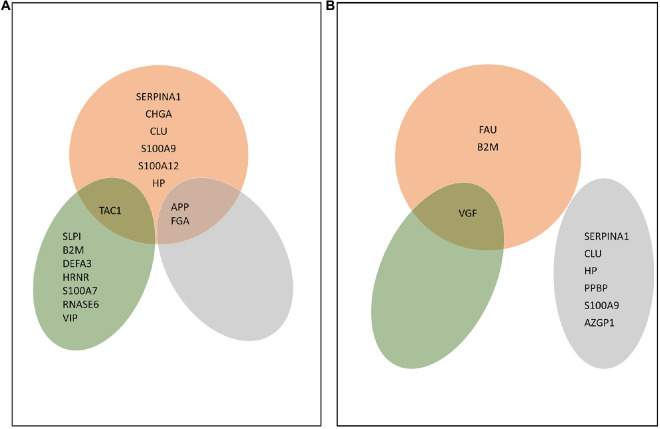
AMPs characteristic of Alzheimer’s disease (AD) in the brain, CSF, and blood. **(A)** AMPs whose amount increased in a statistically significant manner in AD compared with the control. **(B)** AMPs whose amount dropped in a statistically significant manner in AD compared with the control. The Venn diagrams shows the proteins corresponding to each group. Red color shows AMPs found in brain, green refers to AMPs identified in CSF, while gray shows AMPs found in blood. The proteins are shown according to their gene names.

We further investigated the AMPs whose change between the AD and control groups was statistically significant (Figure 3).

In the brain, the increased amount of nine AMPs was characteristic of AD. These were APP, SERPINA1, CHGA, CLU, FGA, S100A9, S100A12, HP, and TAC1 (Figure 3A). There were three AMPs, the FAU, VGF, and B2M, whose amount decreased in AD. In the CSF, SLPI, B2M, DEFA3, HRNR, TAC1, S100A7, RNASE6, and VIP were detected in higher, while VGF in lower amounts in AD.

In the blood, the amount of APP and FGA increased, while the amount of SERPINA1, CLU, HP, PPBP, S100A9, and AZPI decreased in AD (Figure 3B).

**FIGURE 4:**
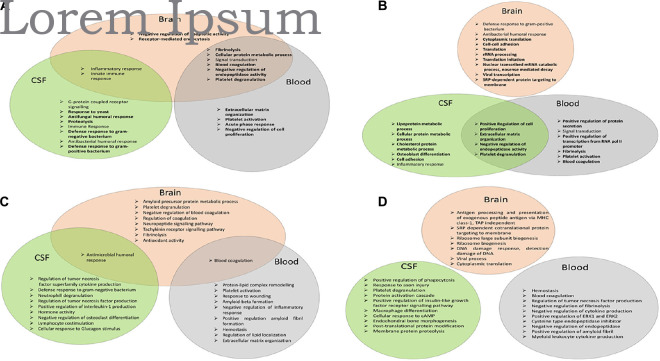
Functional analysis of the network of AMPs characteristic of AD. **(A)** Venn diagram of the top 10 enriched GO terms according to DAVID for AMPs increased in the brain, CSF, or blood. **(B)** Venn diagram of the top 10 enriched GO terms according to DAVID in case of AMPs decreased in the brain, CSF, or blood. **(C)** Venn diagram of the top 10 enriched GO terms according to ClueGO for AMPs increased in the brain, CSF, or blood. **(D)** Venn diagram of the top 10 enriched GO terms according to ClueGO for AMPs lowered in the brain, CSF, or blood.

The network of AMPs characteristic of AD was generated, and due to the relatively low number of AMPs, during network generation, the 50 first shells of interactors were also considered (Figure 4 and Supplementary Figure 3).

For AMPs increased in AD, some of the GO functions enriched in the brain were similar to those enriched in the overall AMP network (Figure 1A): inflammatory response, innate immune response, and signal transduction. The new functions enriched were related to blood coagulation (fibrinolysis, platelet degranulation, and blood coagulation), cellular metabolic process, endocytosis, and negative regulation of endopeptidase activity and of apoptotic activity (Figure 4A). In the CSF, most of the enriched GO functions were related to antimicrobial defense, immune response, inflammation, and G-protein receptor signaling pathway, as seen in the overall AMP network. The new functions enriched in CSF were the proteolysis and the response to yeast. Regarding the enriched functions characteristic of AMPs identified in the blood, the blood coagulation (fibrinolysis, platelet activation, and degranulation), acute phase response, extracellular matrix organization, signal transduction, cellular protein metabolic process, and negative regulation of endopeptidase activity and of cell proliferation should be mentioned.

The inflammatory and innate immune response functions were common between the brain and the CSF, while the functions related to blood clotting, the negative regulation of endopeptidase activity, signal transduction, and cellular protein metabolism were the functions common between the brain and the blood (Figure 4A).

In case of the AMPs decreased in AD, the GO functions in the brain were related to RNA metabolism, translation, viral transcription, cell adhesion, and antibacterial response, and all functions except the last were new functions characteristic of the brain (Figure 4B). In CSF, a large variety of enriched GO functions could be observed: functions related to lipid (lipoprotein and cholesterol) metabolism, cell adhesion, osteoblast differentiation, regulation of cell proliferation and of endopeptidase activity, platelet degranulation, inflammation, and cell adhesion. In blood, GO functions related to blood coagulation, signal transduction, protein secretion, regulation of endopeptidase activity and of cell proliferation, transcription, and extracellular matrix organization were more dominant (Figure 4B). The regulation of cell proliferation and of endopeptidase activity, the extracellular matrix organization, and the platelet degranulation functions were common in both blood and CSF.

The DAVID analysis provides a robust examination of the GO functions. In order to have more information on the functional enrichment, the GO analysis with ClueGO was also considered. Besides the functions identified by DAVID in the case of proteins increased in AD, the amyloid precursor protein metabolic process, neuropeptide and tachykinin signaling pathway, and antioxidant activity are shown to be characteristic of the brain. In the blood, the regulation of lipid localization, protein–lipid complex remodeling, amyloid beta formation, and response to wounding were considered as new enriched GO functions, while the cellular response to glucagon stimulus and regulation of osteoclast differentiation were considered as new GO functions in CSF (Figure 4C). Regarding the analysis of the proteins decreased in AD, the additional functions to those identified by DAVID were the antigen processing and DNA damage response in the brain, the regulation of amyloid fibril formation in the blood, and the response to axon injury, regulation of insulin-like growth factor, cellular response to cAMP, post-translational protein modification, and membrane protein proteolysis in the CSF, when running ClueGO (Figure 4D).

In order to see which proteins might have the highest importance in the network of AMPs, we aimed to identify the main hub proteins. For this reason, we examined the protein–protein interaction network, and calculated the betweenness centrality and the degree of distribution (Supplementary Figure 4). In line with previously published data, we found APP as the main hub protein in the network characteristic of the brain (Jiao et al., 2009; Rao et al., 2013).

**FIGURE 5:**
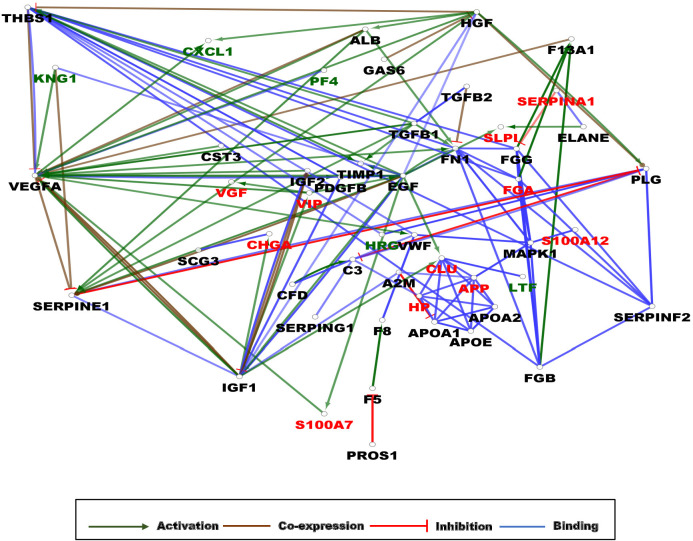
Gene interaction network (GIN) of AMPs characteristic of AD. The circles represent a gene/protein and the lines indicate interactions. The lines with arrow represent activation, blocking lines represent inhibition, simple lines represent protein–protein interaction. Line color indicates the type of interaction: green color refers to activation, red color to inhibition, blue color binding, whereas brown color shows co-expression. The red color of protein label indicates AMPs whose amount changed in a statistically significant manner between AD and control samples, the black color shows their interacting partners with no AMP activity, while the green color indicates interactors with AMP activity. On all panels the proteins are labeled with their gene name. The higher resolution images of the individual networks are presented in Supplementary Figure 5.

Taking into account the fact that no considerable differences could be observed in the gene interaction networks of AMPs present in the UDAMP Database and the ones observed in the brain, CSF, and blood, in the next step, we were eager to examine the possible differences in gene interaction networks in case of AMPs characteristic of AD (Figure 5 and Supplementary Figure 5).

As expected from the examination of protein–protein interaction network analysis, the central role of APP as target of activation by ADAM10, PSEN1, and BACE1 could be demonstrated (Supplementary Figure 5A). The activation of F13B by FGA, FGB, and FGG was also observed, and the inhibitory network involving fibrinogen alpha, beta, and gamma chains, thrombin, factor XIII, SERPINC1, SERPINF2, SERPIND1, and SERPINA10 could be identified (Supplementary Figure 5A). Regarding the AMPs decreased in the brain, the cluster of ribosomal proteins interacting with FAU could be observed (Supplementary Figure 5B). The gene interaction network analysis in CSF showed a complex picture: in the case of AMPs increasing in AD, the NFKB1 and PIK3CA interacting proteins were the main sites of multiple activation and inhibition (Supplementary Figure 5C), while in the case of AMPs decreasing in AD, the IL6 was the main site of regulation (Supplementary Figure 5D).

In the blood, the observed situation was completely different; the regulatory network involving different forms of fibrinogen, SERPINs, and thrombin observed in the case of the brain as well, was linked through the FN1 and MAP kinases to the regulatory network involving mainly activations having FAM20C in the center of the activations. Other highly regulated proteins were IL6 and CDH2 (Supplementary Figure 5E). Considering the regulatory networks involving AMPs whose amount diminished in AD, the TIMP1, SERPINE1, THBS1, and VEGFA were shown as the main sites of regulatory activities involving mainly activations (Supplementary Figure 5F).

In order to be able to handle together the data, the gene interaction network of all proteins changed in AD regardless of the direction of change (increase or decrease), and the site of identification (brain, CSF, or blood) was examined.

On the network, three clusters could be observed: an activation cluster having as hub the VEGFA and two clusters of interacting proteins. In one of the clusters, the APP was the binding partner for apolipoproteins APOA1, APOA2, and APOE and also for CLU, HP, and MAPK1, while in the other cluster, proteins having a role mainly in blood clotting such as FGA, FGB, FGC, FNN, SERPINF2, and THBS1 could be observed. From a mechanistic point of view, the main hub protein was shown to be VEGFA, followed by EGF, IGF1, SERPINE1, and FNN, as proteins activated or inhibited by many other proteins (Figure 5).

## Discussion

The cardinal role of APP in the pathophysiology of AD was investigated extensively, and according to the so-called amyloid hypothesis, the Aβ generated from the cleavage of APP has a fundamental role in the development of the disease (Yun-wu et al., 2011). It is also known from the scientific literature that bacterial or viral infections can lead to the appearance of the disease, and the activation of the immune system is required for the development of AD (Kumar et al., 2016; Venegas et al., 2017; Dominy et al., 2019; András et al., 2020) suggesting a complex phenomenon lying behind the appearance of the symptoms. A direct link between the Aβ and the antimicrobial response was demonstrated by different groups, showing that Aβ peptides participate in killing the bacteria (Kagan et al., 2012) and aggregated Aβ helps the immobilization of microbes (Kumar et al., 2016). It was shown that Aβ is able to interact with the viral coat proteins acting against influenza strains and herpes simplex virus, being as effective antiviral agent as acyclovir against herpes simplex virus 1 (Lukiw et al., 2010; White et al., 2014; Bourgade et al., 2016).

In this study, our aim was to investigate the presence and possible role of AMPs in AD. AMPs being part of the innate immune system modulate the immune reactions and at the same time are directly involved in diminishing pathogens either by killing them or by preventing their growth (Wiesner and Vilcinskas, 2010). We wanted to know which AMPs were already identified by proteomics techniques in the brain, blood, and CSF and also which of them might have a role in the pathophysiology of AD. In order to investigate this question, first, we have collected all available human AMPs by generating the UDAMP Database. The database currently contains 186 AMPs (Table 2), and it is available at Figshare https://doi.org/10.6084/m9.figshare.13590083.v2.

The network analysis of all human AMPs surprisingly defined only three clusters of interacting proteins. Cluster 1 contained the highly interconnected network of defensins having DEFA4 as a hub providing the connection with Cluster 2. Cluster 3 was another highly interconnected cluster containing cytokines and some other AMPs such as neuropeptides (VIP, NPY, VGF, etc.), APP, blood coagulation factors (F2, FGA, SERPINA1), etc. The connection between Clusters 1 and 3 was made through the Cluster 2, which contained fewer number of AMPs compared with the other two clusters. The hub proteins responsible for the connection of Clusters 2 and 3 were CLU and CCL25, CCL21 and B2M. The AMPs functioning as hub proteins in the network were APP, DEFA4, CXCL1, CXCL10, KNG1, PPBP, POMC, PF4, etc., while the most relevant biological functions were related to defense, innate immune system, and signal transduction. The network analysis of human AMPs provided background data involving all known human AMPs and recapitulated the information presented in the scientific literature regarding their functions and roles in human organisms (Yuping and Gallo, 2009; Wiesner and Vilcinskas, 2010; Chessa et al., 2020).

Among the human AMPs listed in the UDAMP Database, 46 were found in the brain, 46 in the CSF, and 75 in the blood. It was observed that the set of AMPs identified in each sample type overlapped but were not identical. Regarding the network analysis, there were some particular characteristics to each sample type; however, regarding the enriched GO functions, mainly, the general AMP functions related to antimicrobial defense, activation of immune system, and blood coagulation were characteristic of AMPs identified in the brain, CSF, or blood.

Regarding the proteins showing a statistically significant change in AD, the picture was different. The level of nine AMPs was shown to be increased in a statistically significant manner in the brain, and two of the AMPs were characteristic for the blood as well. These two proteins (FGA and APP) might have diagnostic potential in AD from blood, and there are different examination methods available for the utilization of FGA and APP as biomarkers (Irizarry, 2004; Joung Wook et al., 2007; Cortes-Canteli et al., 2012). According to our data and the data from the scientific literature, APP was shown to be the major hub protein with the highest number of interactions in AD (Jiao et al., 2009; Rao et al., 2013). Regarding the CSF, there was overlap only in the case of TAC1 with the AMPs increased in the brain and no overlap with AMPs increased in the blood. However, when the AMPs showing a statistically significant reduction in AD were examined, four AMPs elevated in the brain decreased in the blood (CLU, HP, S100A9, SERPINA1). A high level of CLU (or ApoJ) and HP was already associated with AD, but their elevated levels were measured not only in the brain but in the blood as well (Spagnuolo et al., 2014; Song et al., 2015; Gupta et al., 2017; Foster et al., 2019). Taking into account that in the different experiments, the blood collection time was different relative to the onset of AD, most probably more research is required with standardized sample collection times to clarify the situation regarding the dynamics of the change in the level of these proteins in the blood. The situation of B2M and FAU appeared to be similar. B2M increased in the CSF, while it decreased in the brain. Besides B2M, FAU’s level dropped in the brain and of VGF in both brain and CSF. The data published in the scientific literature present elevated B2M and FAU levels in the brain and propose them as potential targets for AD therapy (Smith et al., 2015; Matthes et al., 2018; Li et al., 2020). Regarding TAC1 and VGF, our data are in accordance with the previously published data: neuropeptides derived from TAC1 exert a neuroprotective role in AD (Chen et al., 2019), and the VGF was discovered in mouse model as a key driver gene/protein. It was shown that the increase in VGF level could alleviate the memory impairment symptoms in mice (Beckmann et al., 2020). It was demonstrated with network analysis that a progressive reduction in the level of VGF along with other proteins was characteristic of asymptomatic AD and AD (Seyfried et al., 2018).

Besides those aforementioned AMPs, there are other AMPs that either significantly increase or decrease in patients with AD. CHGA was shown to play a role in activating microglia, which can release neurotoxins, leading to the apoptosis of neurons (Sathe et al., 2020). Some of the other AMPs were already linked to AD; S100A7 is a recently discovered potential biomarker for AD (Qin et al., 2009; Fleming et al., 2012), and PPBP and AZGP1 were shown to be downregulated in the serum of patients with AD (Shen et al., 2017); our study confirms their downregulation in CSF. The increase in VIP can be interpreted as an attempt to prevent the worsening of the neurodegeneration process, as far as VIP has an inhibitory effect on the activation of microglial cells, therefore hindering the release of more inflammatory cytokines (Delgado and Ganea, 2003).

Examining the functions not related to the innate immune system of the differentially expressed AMPs, it was found that most of the proteins have protective roles, such as the antioxidant HP, which is responsible for hem binding (Wassell, 2000) and CLU, which inhibits the formation of amyloid fibrils and prevents stress-induced aggregation of proteins (Yerbury et al., 2007). The fibrinogen A (FGA), along with SERPINA1 and platelet basic protein (PPBP), has a role in hemostasis (Rau et al., 2008; Smyth et al., 2009; Sørensen et al., 2011) preventing blood loss. The FAU, or 40S ribosomal protein S30, has a role in DNA repair and function (DNA damage), which seemed to lower in AD brain (Lee et al., 2011).

Regarding the network analysis of AMPs altered in AD, our data give further evidence for the importance of inflammation and innate defense mechanisms in the development of the disease. Beside these general functions, alterations affecting protein post-translational modification, which might be related to hyperphosphorylation of tau (or of other proteins) (Tan et al., 2015), ubiquitination (Measho et al., 2018), glycosylation (Wang et al., 2017), or citrullination (Deolankar et al., 2019) of brain proteins characteristic of AD could be demonstrated. The dietary excess and impairment of lipid metabolism, along with insulin intolerance and the lack of neurotrophic factors, can lead to the cognitive decline characteristic of AD (Schindowski et al., 2008; de la Monte, 2009; Allen et al., 2011; Hu et al., 2013; Dharshini et al., 2019; Mitra et al., 2019; Kellar and Craft, 2020). Our data give further evidence on the alterations of lipoprotein metabolism and cholesterol metabolism observed when studying the CSF samples originating from patients with AD. With network analysis, we could demonstrate the importance of hemostasis as well. The role of blood clotting in the development of AD was studied, and it was shown to be compromised (Suidan et al., 2018). Some of the clotting factors may destroy the synapses offering in this way new potential targets for diagnosis and therapeutic intervention (Merlini et al., 2019). The alterations related to GO functions such as APP metabolism, amyloid fibril formation, inflammation, post-translational protein modification, translation, antimicrobial defense, hemostasis, response to axonal injury, regulation of IGFR signaling, cellular response to cAMP, etc., suggest the central role of AMPs in the development of the disease.

Studies aiming to understand at network level the pathophysiological phenomena leading to the development of AD and also to the worsening of the disease have identified modules of proteins having a role in the pathophysiology of the disease. As far as we were concentrating only on the AMPs, the input for these types of studies are different from ours allowing only for the comparison of GO functions. Protein modules and proteins related to synapse, mitochondrial function, glucose and carbohydrate metabolism, extracellular matrix, cytoskeleton, and RNA binding/splicing have been linked to AD (Bader et al., 2020; Johnson et al., 2020) being in line with our results regarding the altered functions related to extracellular matrix organization observed in the CSF and the blood and to the modified RNA metabolism observed in the brain and blood. The modifications related to RNA metabolism were demonstrated in AD as RNA-binding proteins, and RNA splicing was shown to be important in the worsening of the symptoms of AD (Johnson et al., 2018). Other groups have identified functions related to proteostasis, RNA homeostasis, immune response, neuroinflammation, synaptic transmission, vesicular transport, cell signaling, cellular metabolism, lipid homeostasis, mitochondrial function, cytoskeleton organization, and myelin–axon interactions as key players in the pathology of AD (Zhang et al., 2018), most of which functions have been demonstrated in our analysis as well.

Based on the composite network analysis, the VEGF was identified as a functional hub protein regulated by the highest number of proteins, and it was demonstrated by other groups to have a role in AD (Mahoney et al., 2019). Similarly, the other hub proteins were identified; the IGF-1 (Westwoo et al., 2014), EGF (Lei et al., 2012; Siddiqui et al., 2012), SERPINE1 (Akhter et al., 2018), and fibronectin (Anna et al., 2009) were linked earlier to aging, neurodegeneration, and AD.

In spite of their relatively low number (0.5–1% of all identified proteins), the important functions described earlier in AD could be demonstrated with the network analysis of AMPs. Our data shed light on the importance of examinations related to AMPs and emphasize the key role of the innate immune system and blood clotting in AD.

Our analysis suggests the deep involvement of AMPs in AD. AMPs can be in this way good candidates for further mechanistic studies aiming to understand their exact role in the complex pathophysiology of AD (Zhong et al., 2020) and may serve as targets for future therapies.

## Data Availability Statement

Publicly available datasets were analyzed in this study. This data can be found here: http://proteomecentral.proteomexchange.org/cgi/GetDataset, PXD009199, PXD008807, PXD012203, PXD004510, PXD005321, PXD000067, PXD014376, PXD00 6122, PXD0014557, PXD007694, PXD012851, PXD008098, and PXD0011482.

## Author Contributions

AK performed the experiments, analyzed the data, and prepared the figures. VD evaluated the data and wrote the manuscript. BK designed and performed the programmatic data acquisition, and wrote and edited the manuscript. ÉC designed the research, supervised the experiments, evaluated the data, and wrote and edited the manuscript. All authors contributed to the article and approved the submitted version.

## Conflict of Interest

The authors declare that the research was conducted in the absence of any commercial or financial relationships that could be construed as a potential conflict of interest.

## Footnotes

1 https://www.uniprot.org/uniprot/

2 ftp://ftp.ncbi.nlm.nih.gov/gene/DATA

3 https://blast.ncbi.nlm.nih.gov/Blast.cgi?PAGE=Proteins

4 https://www.uniprot.org/

5 https://www.ncbi.nlm.nih.gov/genbank/

6 https://www.rcsb.org/

7 https://string-db.org/

8 https://doi.org/10.6084/m9.figshare.13590083.v2
