# Supplementary material for: Construction of Unified Human Antimicrobial and Immunomodulatory Peptide Database and Examination of Antimicrobial and Immunomodulatory Peptides in Alzheimer’s Disease Using Network Analysis of Proteomics Datasets
Source: Front Genet. 2021 Apr 28;12:633050. doi: 10.3389/fgene.2021.633050 (PMC8113759; doi:10.3389/fgene.2021.633050)
Supplement: Supplementary file 2 [file Data_Sheet_1.PDF]

Figure S1: High resolution images of the interaction networks involving human AMPs. A. Enriched top 10 GO terms in case of AMPs present in UDAMP Database. The enriched GO terms are shown on the ”y” axis, while the gene counts on the ”x” axis. **B.** Protein-protein interaction (PPI) network. Each rectangle represents a protein and the line represents the interactions. **C.** Gene interaction network (GIN). Circles represent a gene/protein and the lines indicate interactions. The lines with arrow represent activation, blocking lines represent inhibition, simple lines represent protein-protein interaction. Line colour indicates the type of interaction: green colour refers to activation, red colour to inhibition, blue colour binding, whereas yellow colour shows co-expression. On all panels the proteins are labelled with their gene name.

A

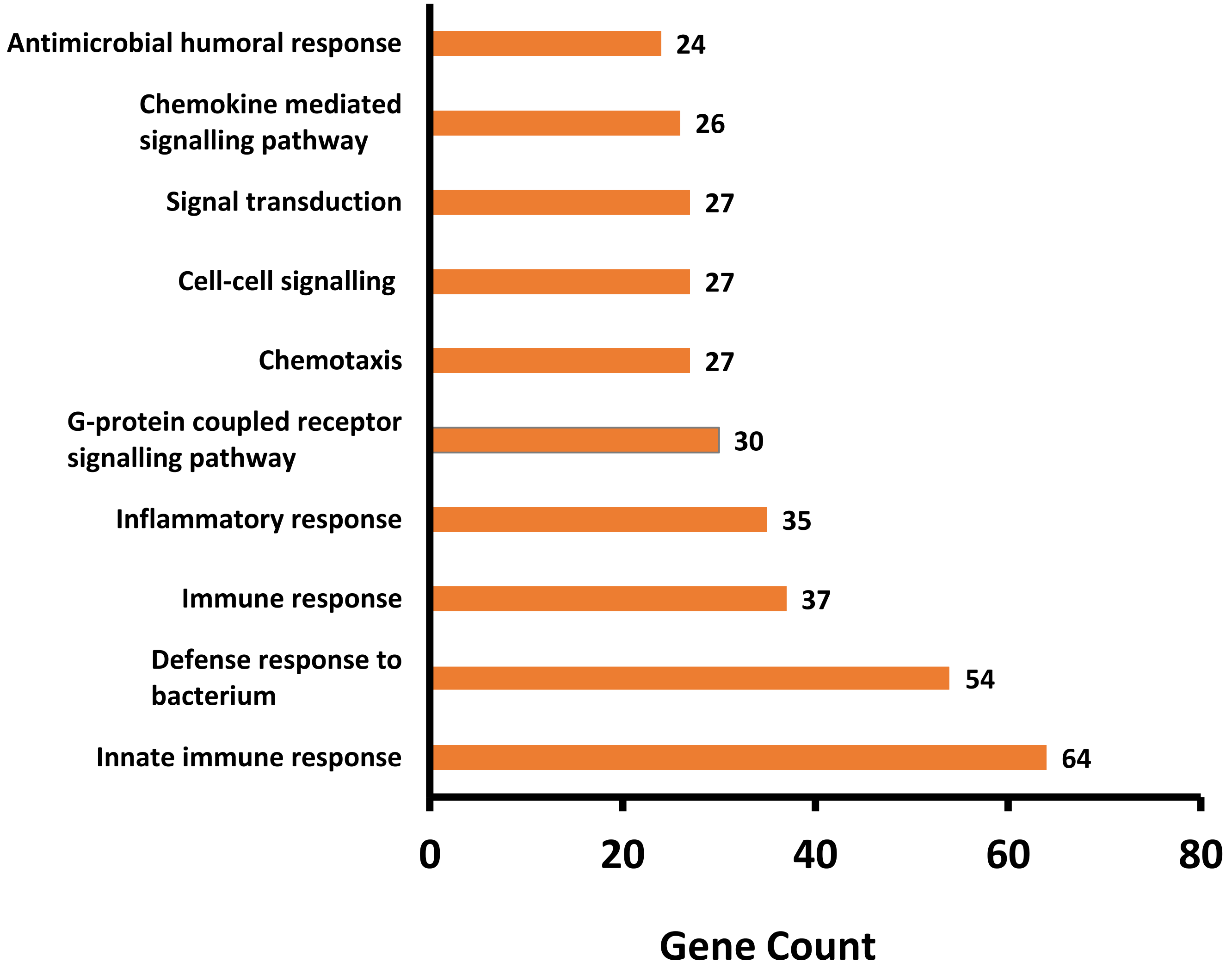
Antimicrobial humoral response
24
Chemokine mediated signalling pathway
26
Signal transduction
27
Cell-cell signalling
27
Chemotaxis
27
G-protein coupled receptor signalling pathway
30
Inflammatory response
35
Immune response
37
Defense response to bacterium
54
Innate immune response
64
0
20
40
60
80
Gene Count

B

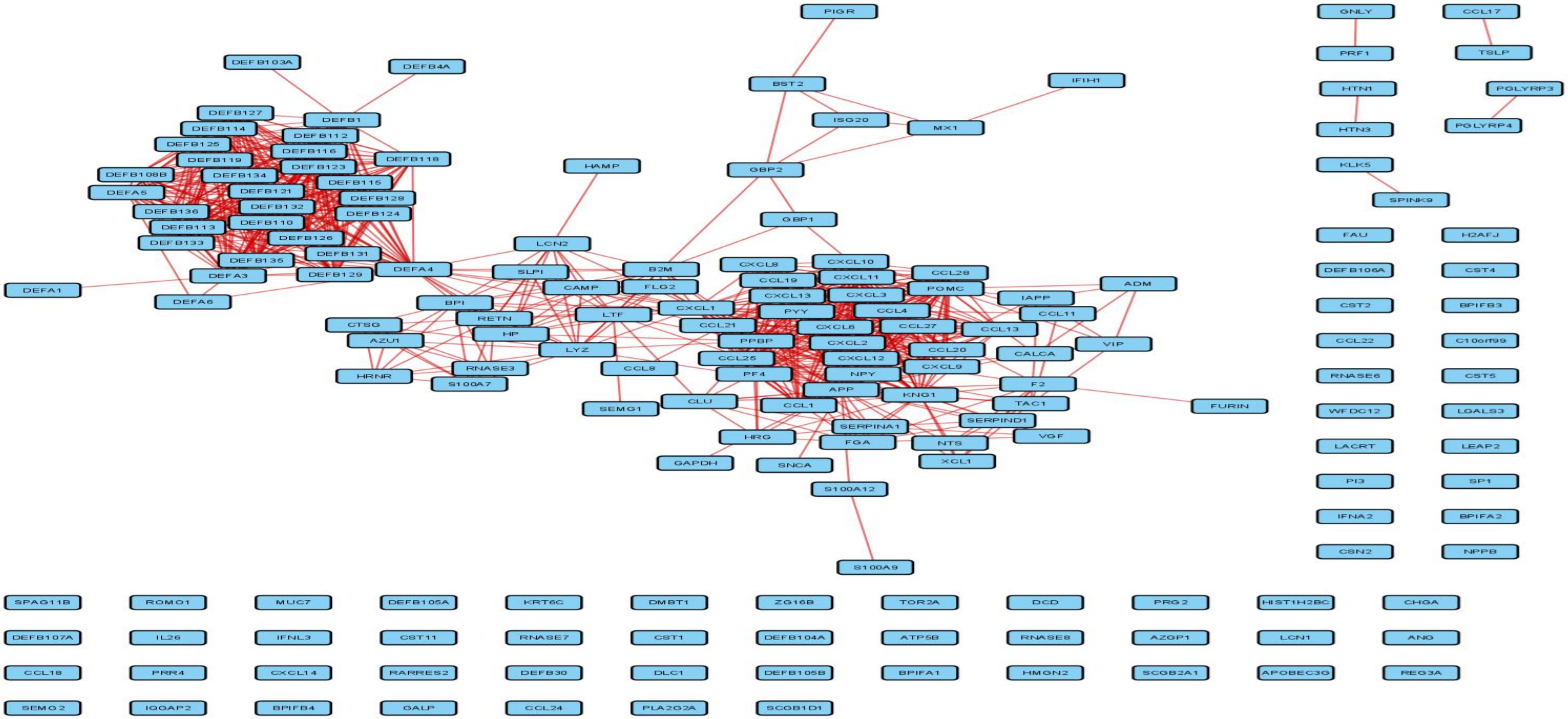
PIGR
GNLY
CCL17
DEFB103A
DEFB4A
BST2
IFIH1
PRF1
TSLP
DEFB127
DEFB1
ISG20
MX1
HTN1
PGLYRP3
DEFB114
DEFB112
HTN3
PGLYRP4
DEFB125
DEFB116
DEFB118
DEFB119
DEFB123
HAMP
GBP2
KLK5
DEFB108B
DEFB134
DEFB115
DEFA5
DEFB121
DEFB128
SPINK9
DEFB136
DEFB132
DEFB124
GBP1
DEFB113
DEFB110
FAU
H2AFJ
DEFB133
DEFB126
LCN2
DEFB131
CXCL10
DEFB135
DEFA4
SLPI
B2M
CXCL8
CCL28
DEFB106A
CST4
DEFA3
DEFB129
CXCL11
DEFA1
CAMP
FLG2
CCL19
POMC
ADM
CXCL13
CXCL3
IAPP
DEFA6
BPI
CXCL1
PYY
CCL4
CST2
BPIFB3
RETN
LTF
CCL21
CCL11
CTSG
CXCL6
CCL27
CCL13
HP
CXCL2
AZU1
PPBP
CCL20
VIP
CCL22
C10orf99
LYZ
CALCA
CCL25
CXCL12
RNASE3
CCL8
CXCL9
HRNR
PF4
NPY
RNASE6
CST5
S100A7
F2
APP
KNG1
CLU
FURIN
TAC1
SEMG1
CCL1
WFDC12
LGALS3
SERPIND1
SERPINA1
HRG
VGF
FGA
NTS
LACRT
LEAP2
GAPDH
SNCA
XCL1
PI3
SP1
S100A12
IFNA2
BPIFA2
CSN2
NPPB
S100A9
SPAG11B
ROMO1
MUC7
DEFB105A
KRT6C
DMBT1
ZG16B
TOR2A
DCD
PRG2
HIST1H2BC
CHGA
DEFB107A
IL26
IFNL3
CST11
RNASE7
CST1
DEFB104A
ATP5B
RNASE8
AZGP1
LCN1
ANG
CCL18
PRR4
CXCL14
RARRES2
DEFB30
DLC1
DEFB105B
BPIFA1
HMGN2
SCGB2A1
APOBEC3G
REG3A
SEMG2
IQGAP2
BPIFB4
GALP
CCL24
PLA2G2A
SCGB1D1

C

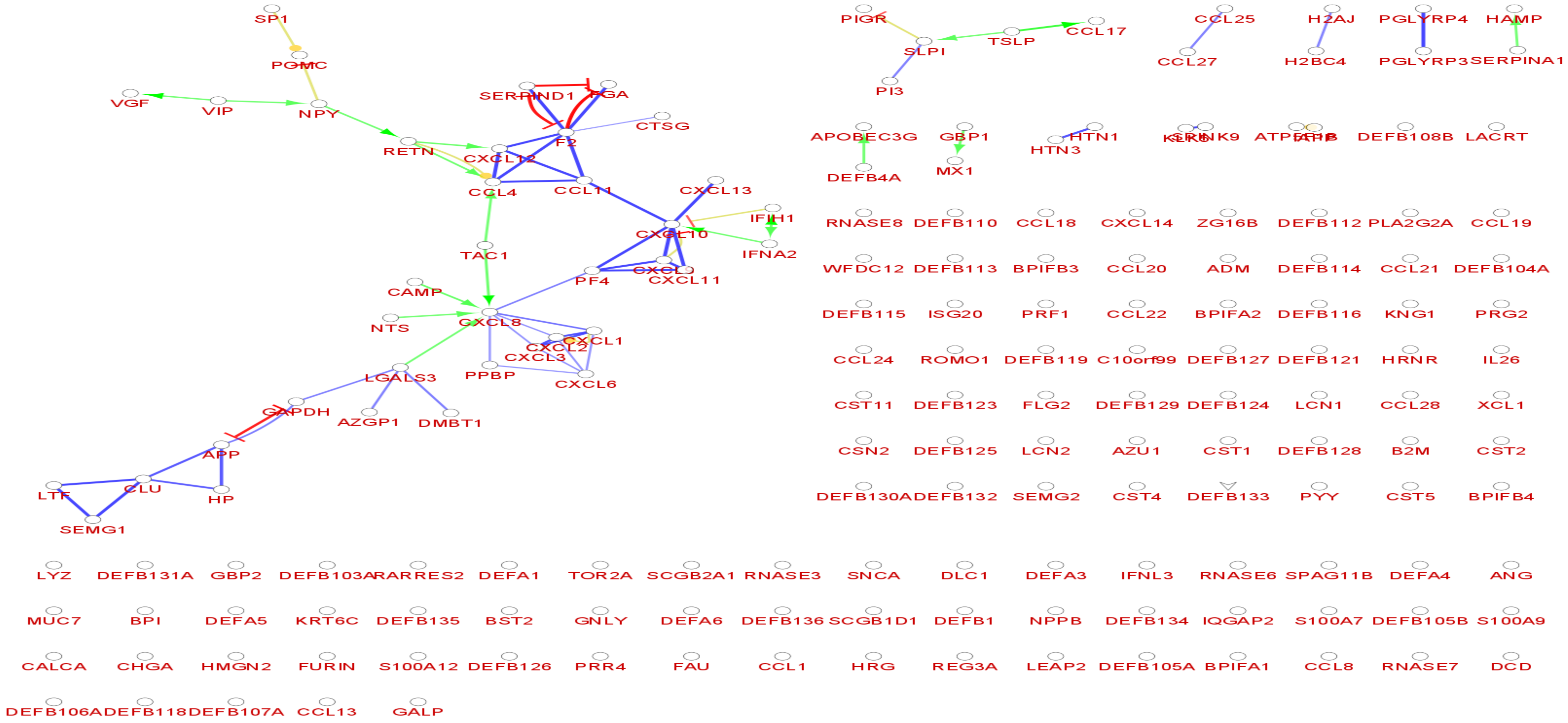

SP1
PGMC
VGF
VIP
NPY
SERPIND1
FGA
CTSG
RETN
CXCL12
F2
CCL4
CCL11
CXCL13
IFIH1
CXCL10
IFNA2
TAC1
PF4
CXCL11
CAMP
CXCL8
NTS
CXCL1
CXCL2
CXCL3
PPBP
CXCL6
LGALS3
GAPDH
AZGP1
DMBT1
APP
LTF
CLU
HP
SEMG1
PIGR
SLPI
TSLP
CCL17
PI3
CCL25
CCL27
H2AJ
H2BC4
PGLYRP4
PGLYRP3
HAMP
SERPINA1
APOBEC3G
DEFB4A
GBP1
MX1
HTN1
HTN3
SPINK9
ATP5F1B
DEFB108B
LACRT
RNASE8
DEFB110
CCL18
CXCL14
ZG16B
DEFB112
PLA2G2A
CCL19
WFDC12
DEFB113
BPIFB3
CCL20
ADM
DEFB114
CCL21
DEFB104A
DEFB115
ISG20
PRF1
CCL22
BPIFA2
DEFB116
KNG1
PRG2
CCL24
ROMO1
DEFB119
C10orf99
DEFB127
DEFB121
HRNR
IL26
CST11
DEFB123
FLG2
DEFB129
DEFB124
LCN1
CCL28
XCL1
CSN2
DEFB125
LCN2
AZU1
CST1
DEFB128
B2M
CST2
DEFB130A
DEFB132
SEMG2
CST4
DEFB133
PYY
CST5
BPIFB4
LYZ
DEFB131A
GBP2
DEFB103A
RARRES2
DEFA1
TOR2A
SCGB2A1
RNASE3
SNCA
DLC1
DEFA3
IFNL3
RNASE6
SPAG11B
DEFA4
ANG
MUC7
BPI
DEFA5
KRT6C
DEFB135
BST2
GNLY
DEFA6
DEFB136
SCGB1D1
DEFB1
NPPB
DEFB134
IQGAP2
S100A7
DEFB105B
S100A9
CALCA
CHGA
HMGN2
FURIN
S100A12
DEFB126
PRR4
FAU
CCL1
HRG
REG3A
LEAP2
DEFB105A
BPIFA1
CCL8
RNASE7
DCD
DEFB106A
DEFB118
DEFB107A
CCL13
GALP

Figure S2. Functional analysis of the networks of proteins identified in AD in the brain, CSF or blood. The enriched GO terms with DAVID and ClueGO, respectively, are shown on the ”y” axis, while the gene counts on the ”x” axis.

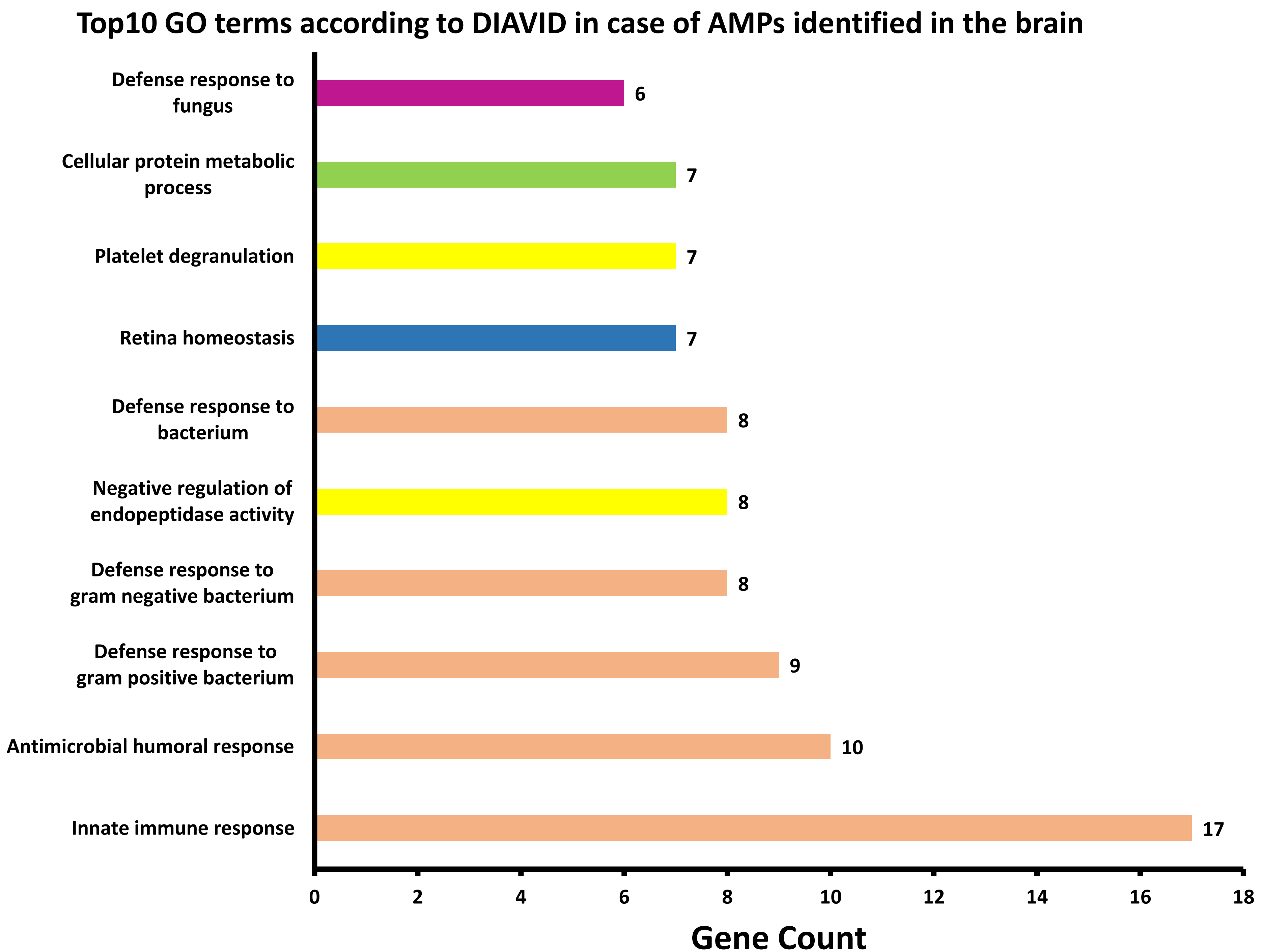
Top10 GO terms according to DIAVID in case of AMPs identified in the brain
Defense response to fungus
6
Cellular protein metabolic process
7
Platelet degranulation
7
Retina homeostasis
7
Defense response to bacterium
8
Negative regulation of endopeptidase activity
8
Defense response to gram negative bacterium
8
Defense response to gram positive bacterium
9
Antimicrobial humoral response
10
Innate immune response
17
0
2
4
6
8
10
12
14
16
18
Gene Count

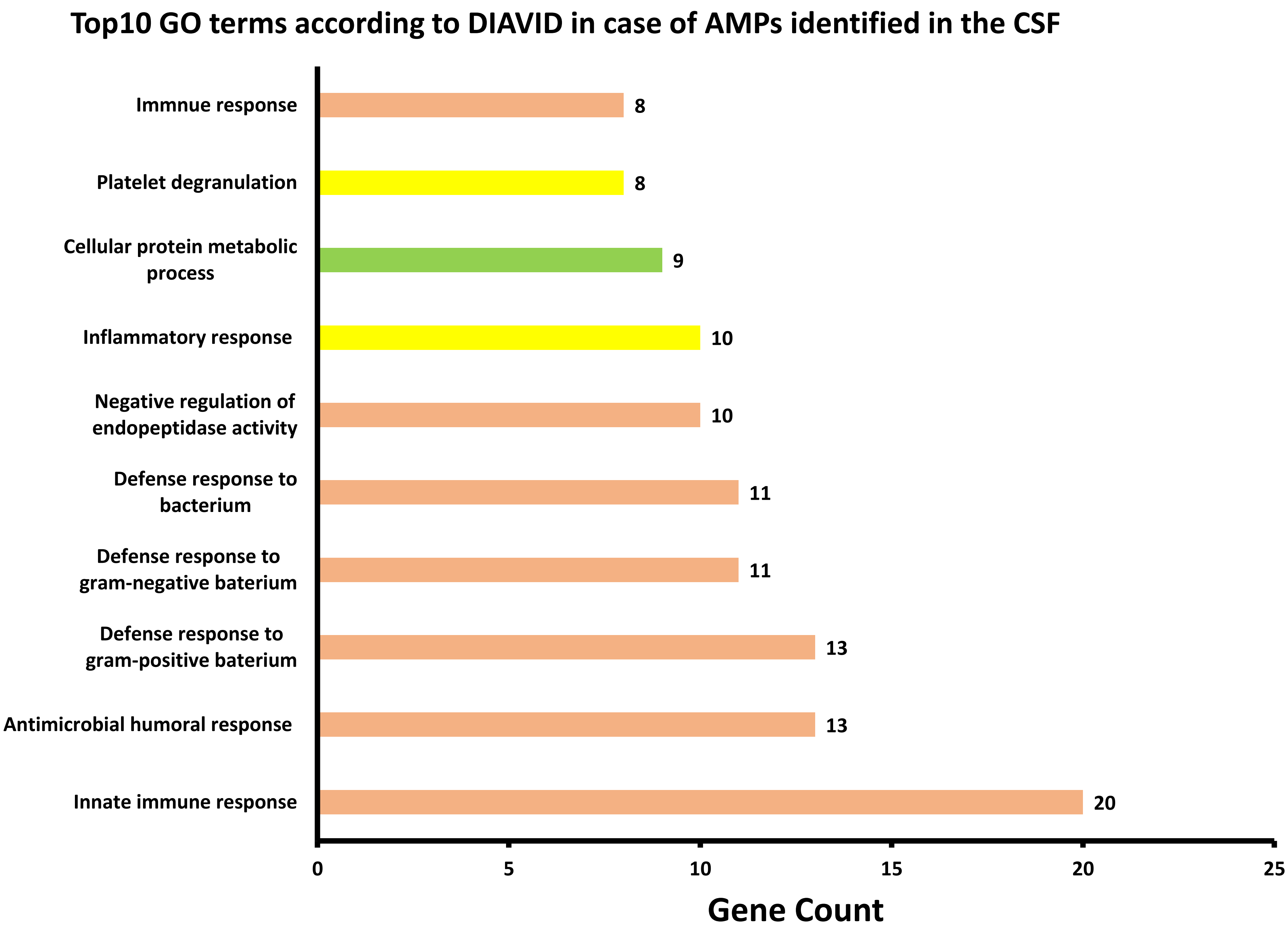
Top10 GO terms according to DIAVID in case of AMPs identified in the CSF
Immnue response
8
Platelet degranulation
8
Cellular protein metabolic process
9
Inflammatory response
10
Negative regulation of endopeptidase activity
10
Defense response to bacterium
11
Defense response to gram-negative baterium
11
Defense response to gram-positive baterium
13
Antimicrobial humoral response
13
Innate immune response
20
0
5
10
15
20
25
Gene Count

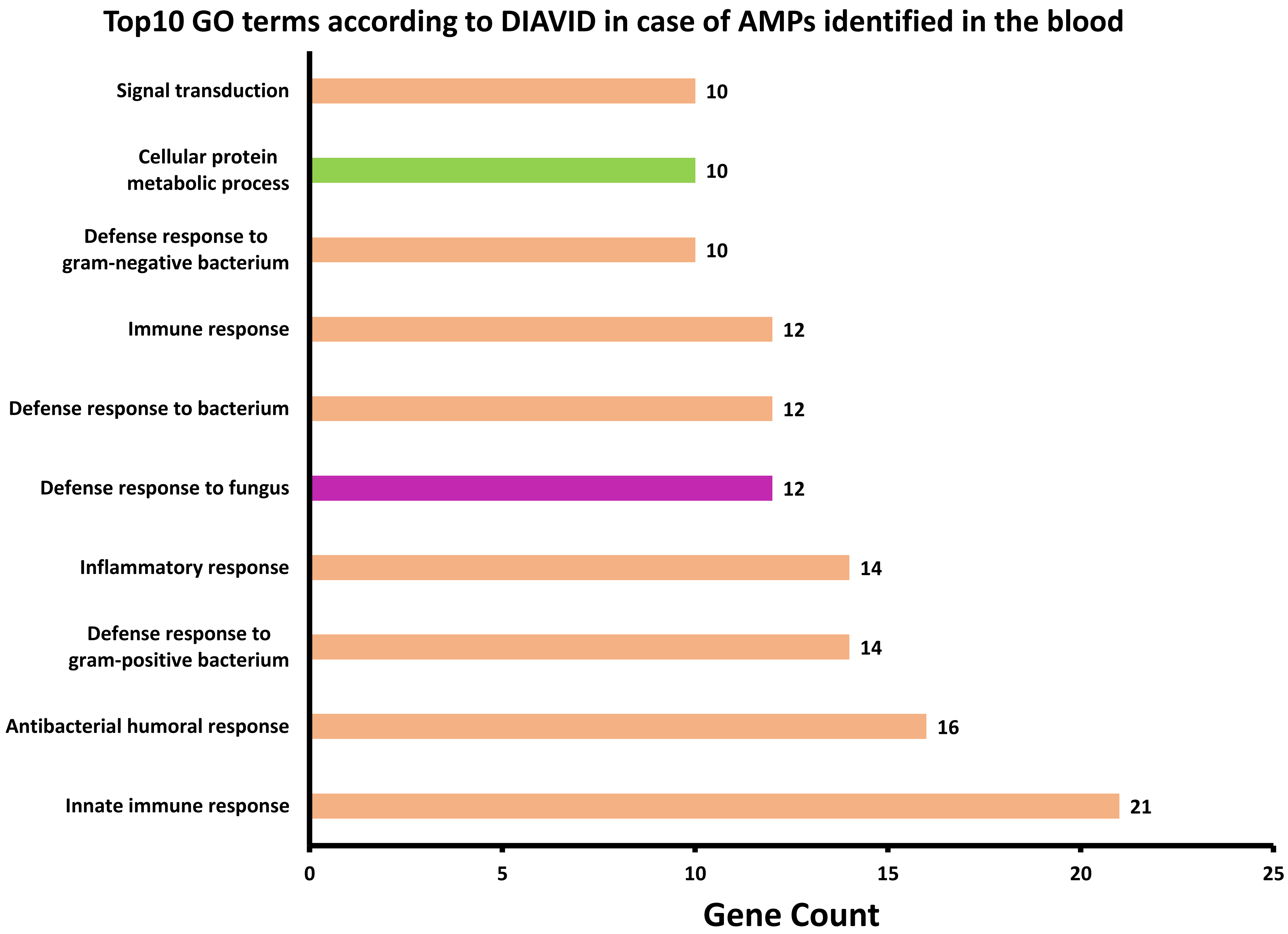
Top10 GO terms according to DIAVID in case of AMPs identified in the blood
Signal transduction
10
Cellular protein metabolic process
10
Defense response to gram-negative bacterium
10
Immune response
12
Defense response to bacterium
12
Defense response to fungus
12
Inflammatory response
14
Defense response to gram-positive bacterium
14
Antibacterial humoral response
16
Innate immune response
21
0
5
10
15
20
25
Gene Count

Top10 GO terms according to ClueGO in case of AMPs identified in the brain

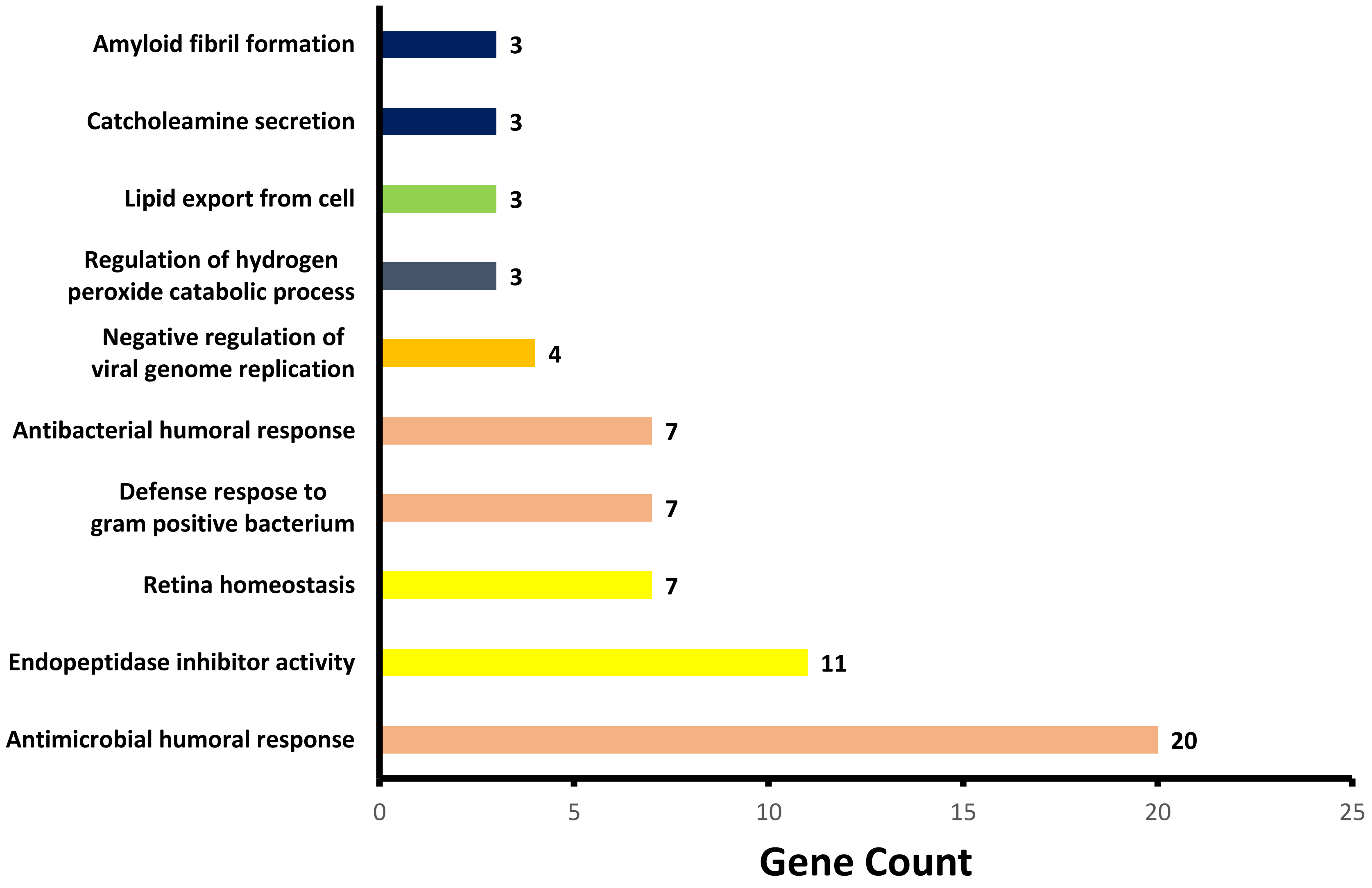

Top10 GO terms according to ClueGO in case of AMPs identified in the CSF

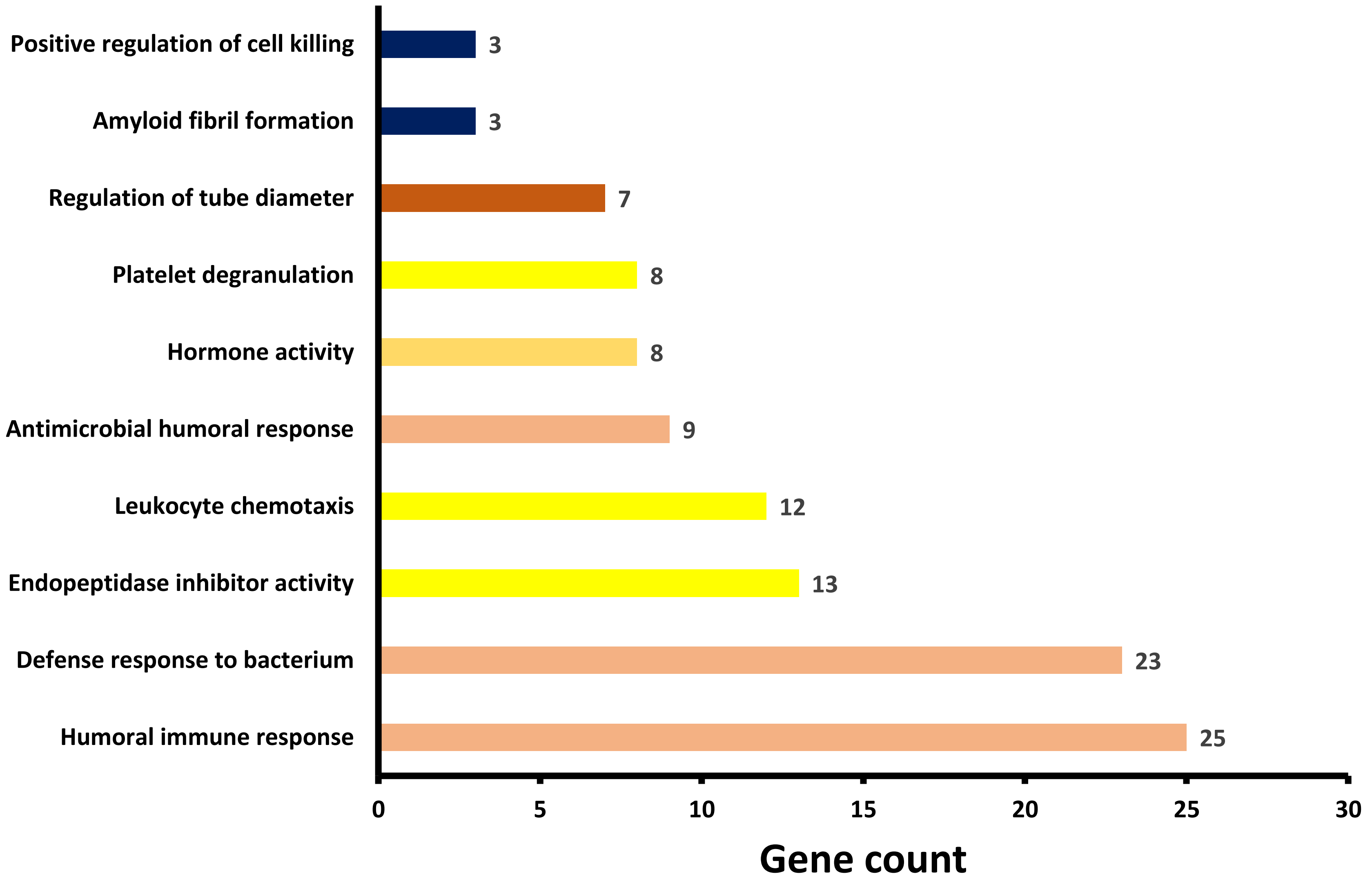

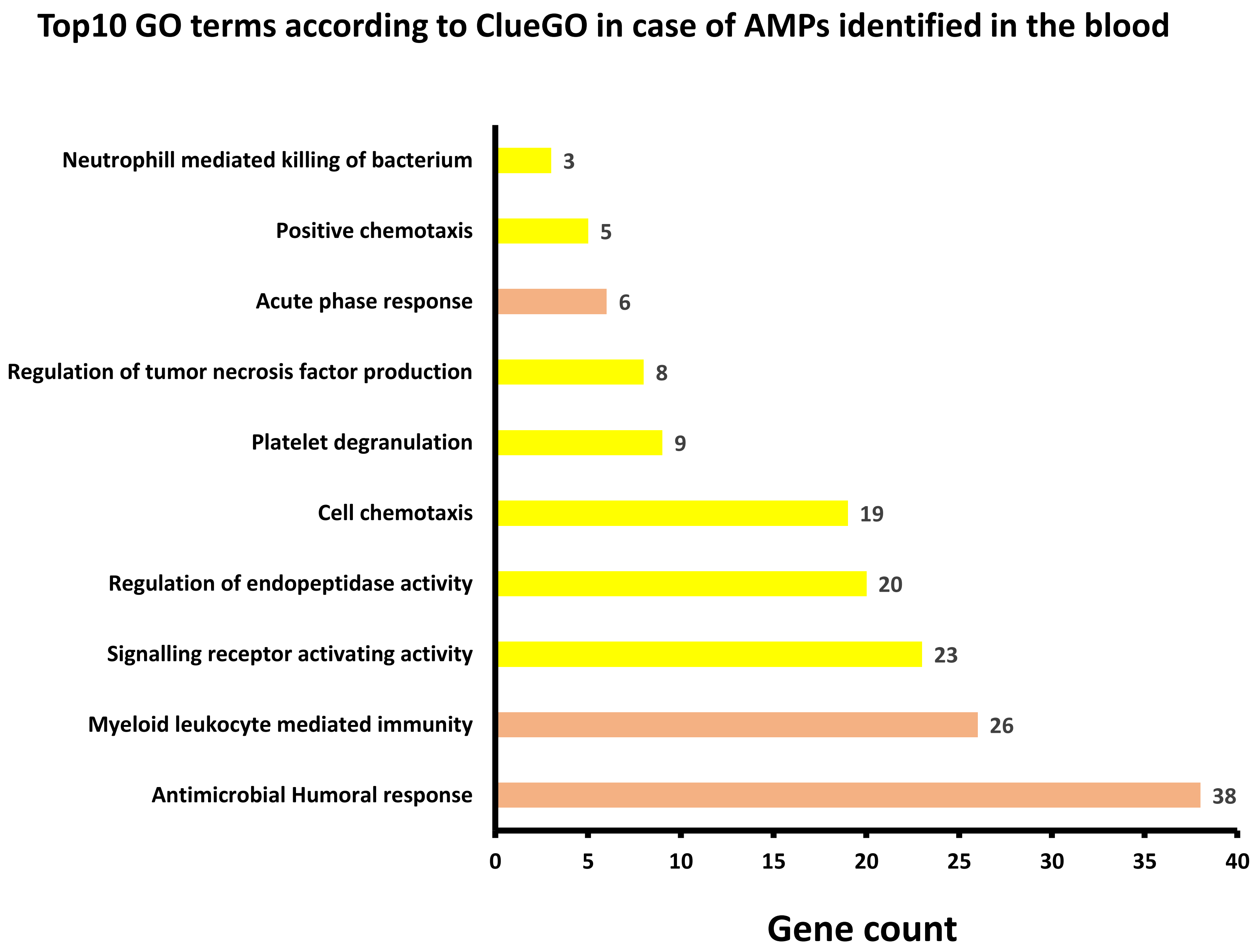
Top10 GO terms according to ClueGO in case of AMPs identified in the blood
Neutrophill mediated killing of bacterium
3
Positive chemotaxis
5
Acute phase response
6
Regulation of tumor necrosis factor production
8
Platelet degranulation
9
Cell chemotaxis
19
Regulation of endopeptidase activity
20
Signalling receptor activating activity
23
Myeloid leukocyte mediated immunity
26
Antimicrobial Humoral response
38
0
5
10
15
20
25
30
35
40
Gene count

Figure S3. Functional analysis of the networks of proteins changed in AD in the brain, CSF or blood. The enriched GO terms with DAVID and ClueGO, respectively, are shown on the ”y” axis, while the gene counts on the ”x” axis.

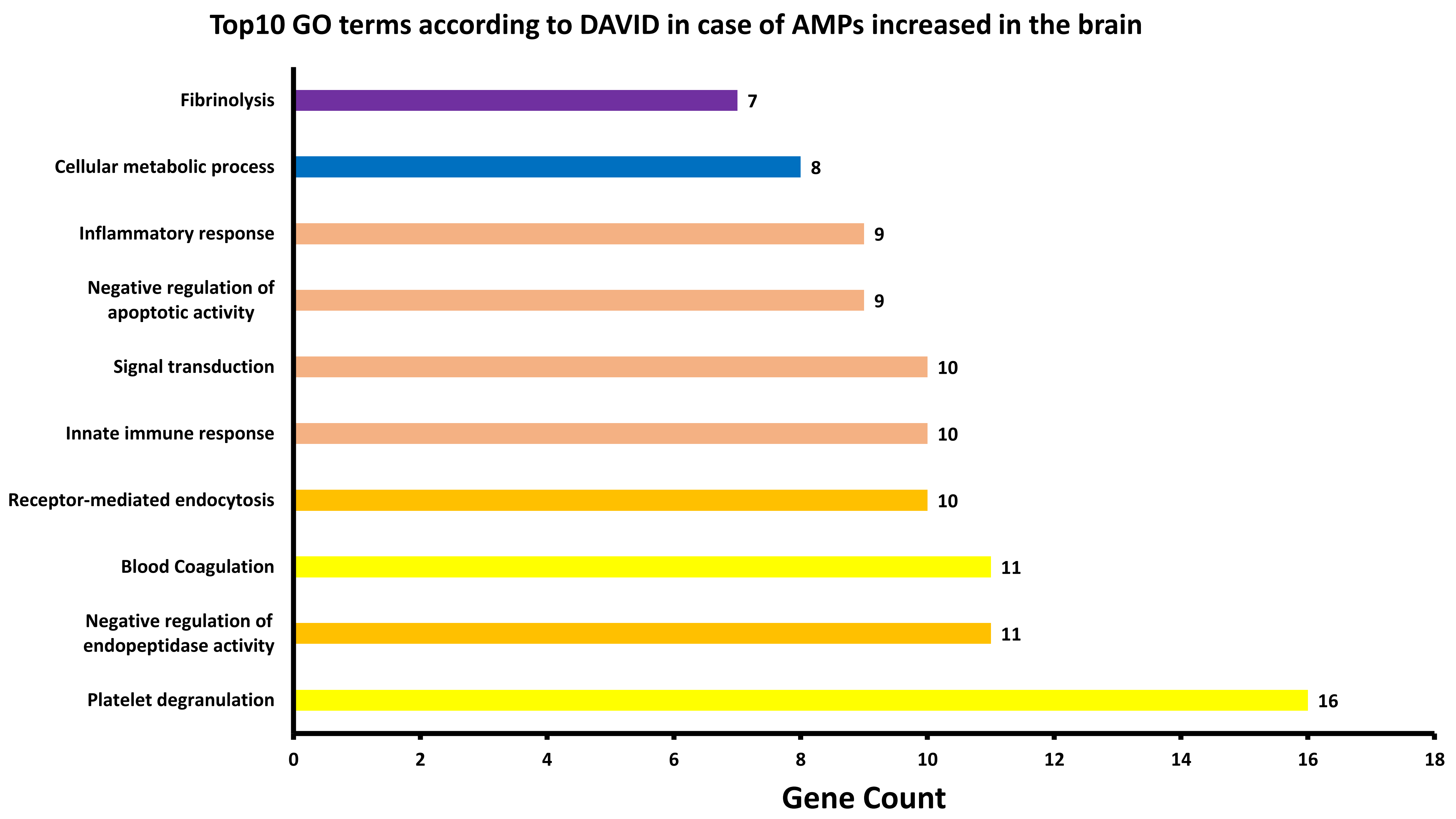
Top10 GO terms according to DAVID in case of AMPs increased in the brain
Fibrinolysis
7
Cellular metabolic process
8
Inflammatory response
9
Negative regulation of apoptotic activity
9
Signal transduction
10
Innate immune response
10
Receptor-mediated endocytosis
10
Blood Coagulation
11
Negative regulation of endopeptidase activity
11
Platelet degranulation
16
0
2
4
6
8
10
12
14
16
18
Gene Count

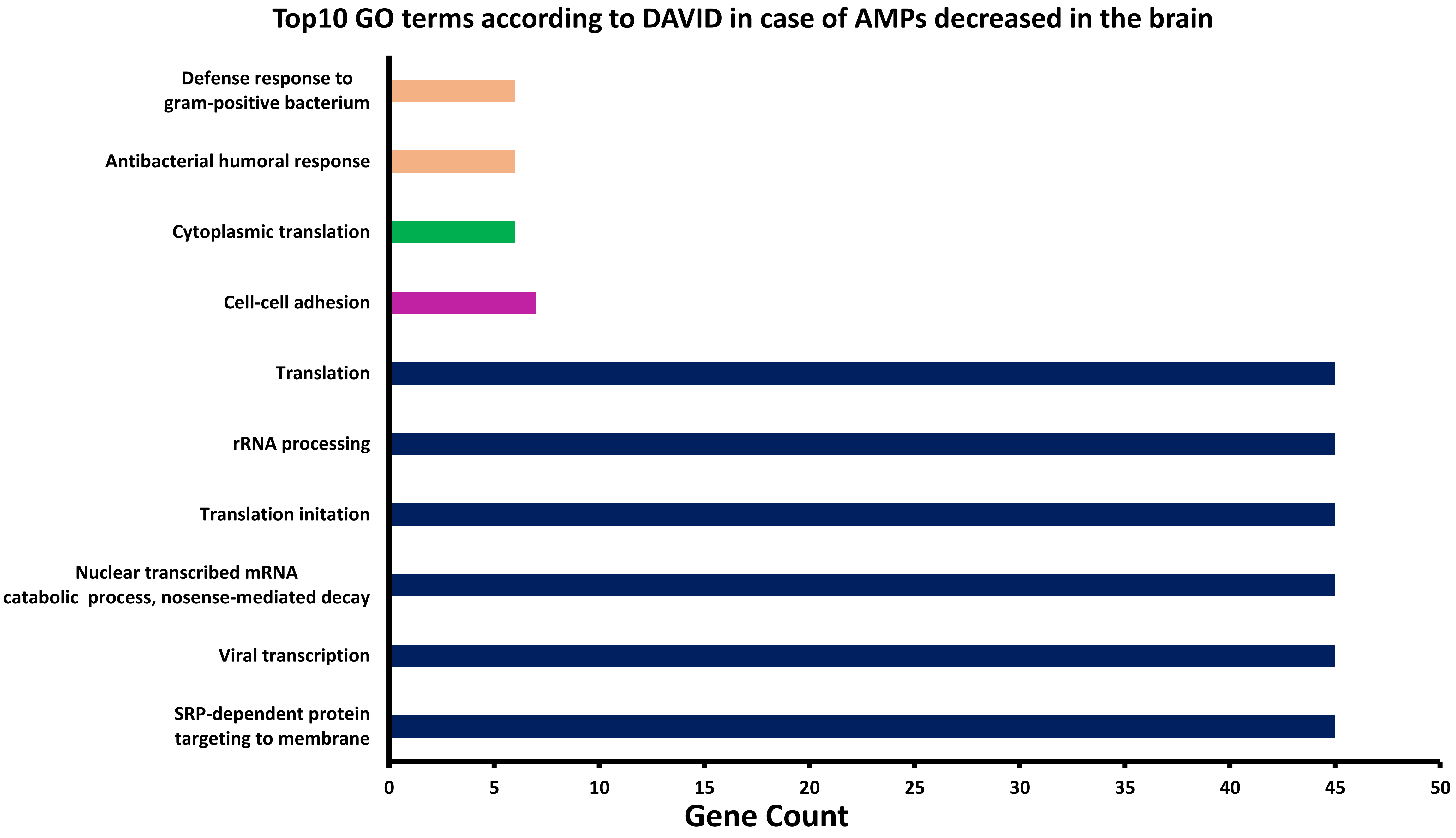
Top10 GO terms according to DAVID in case of AMPs decreased in the brain
Defense response to gram-positive bacterium
Antibacterial humoral response
Cytoplasmic translation
Cell-cell adhesion
Translation
rRNA processing
Translation initation
Nuclear transcribed mRNA catabolic process, nosense-mediated decay
Viral transcription
SRP-dependent protein targeting to membrane
0
5
10
15
20
25
30
35
40
45
50
Gene Count

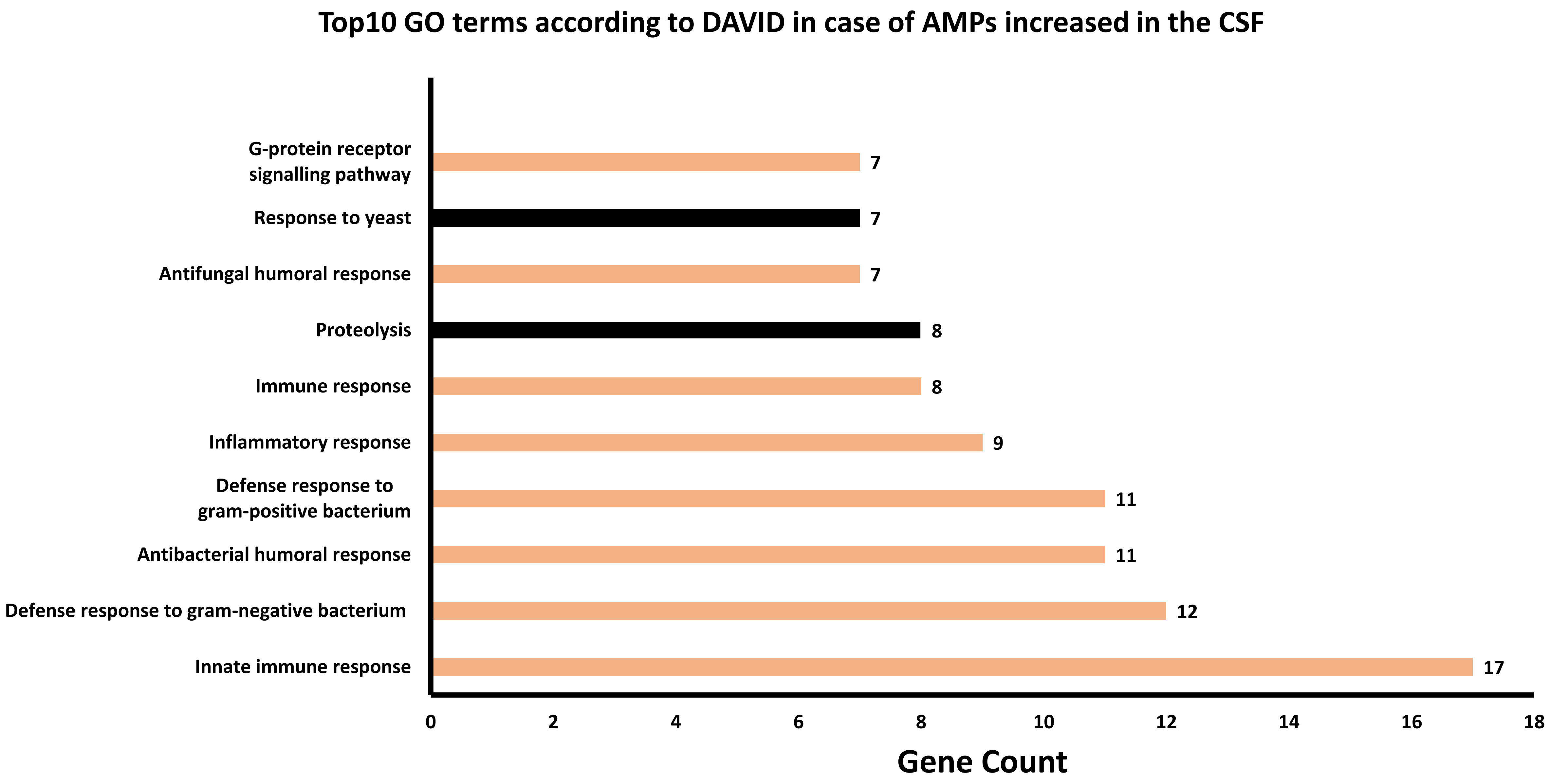
Top10 GO terms according to DAVID in case of AMPs increased in the CSF
G-protein receptor signalling pathway
7
Response to yeast
7
Antifungal humoral response
7
Proteolysis
8
Immune response
8
Inflammatory response
9
Defense response to gram-positive bacterium
11
Antibacterial humoral response
11
Defense response to gram-negative bacterium
12
Innate immune response
17
0
2
4
6
8
10
12
14
16
18
Gene Count

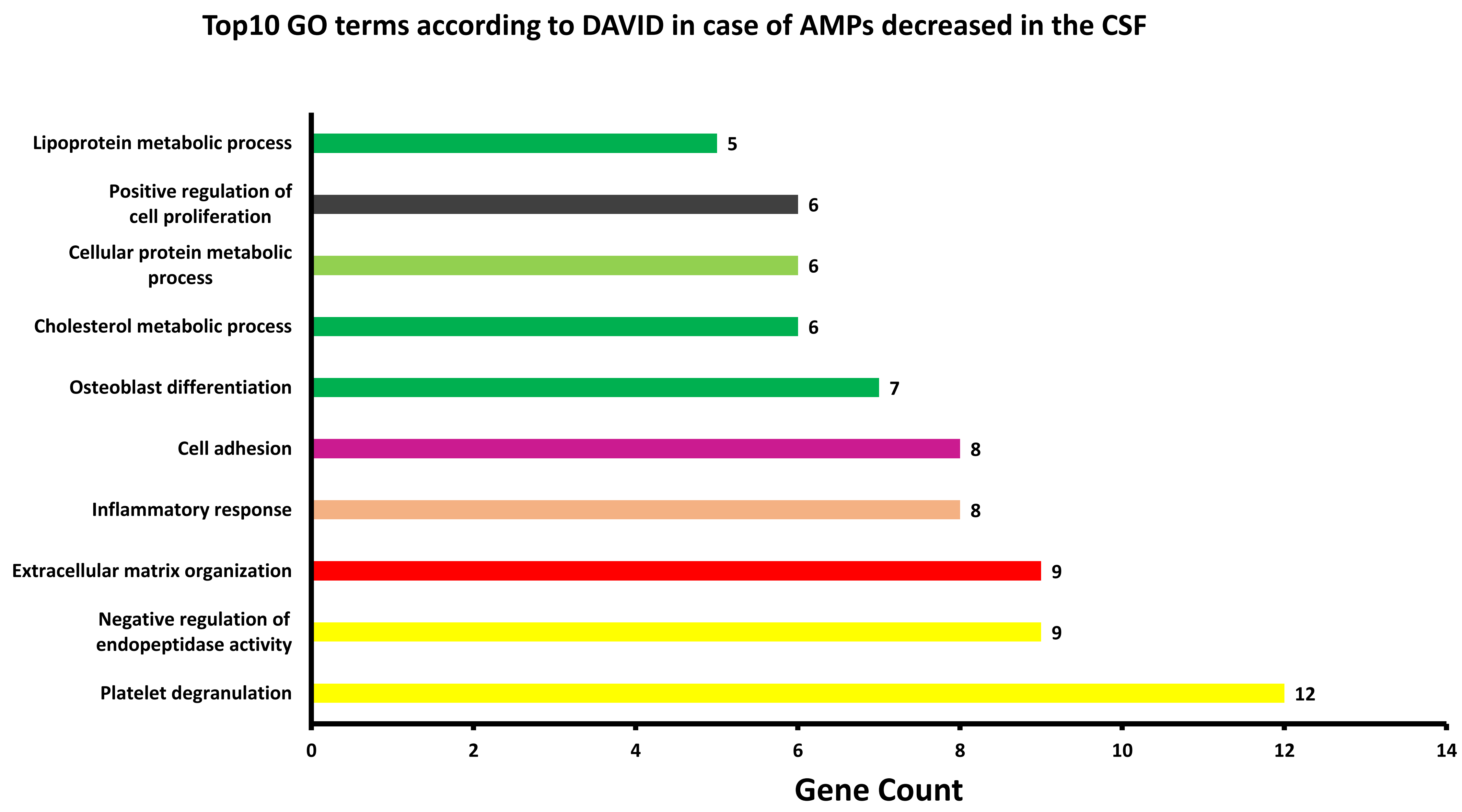

Top10 GO terms according to DAVID in case of AMPs decreased in the CSF
Lipoprotein metabolic process
5
Positive regulation of cell proliferation
6
Cellular protein metabolic process
6
Cholesterol metabolic process
6
Osteoblast differentiation
7
Cell adhesion
8
Inflammatory response
8
Extracellular matrix organization
9
Negative regulation of endopeptidase activity
9
Platelet degranulation
12
0
2
4
6
8
10
12
14
Gene Count

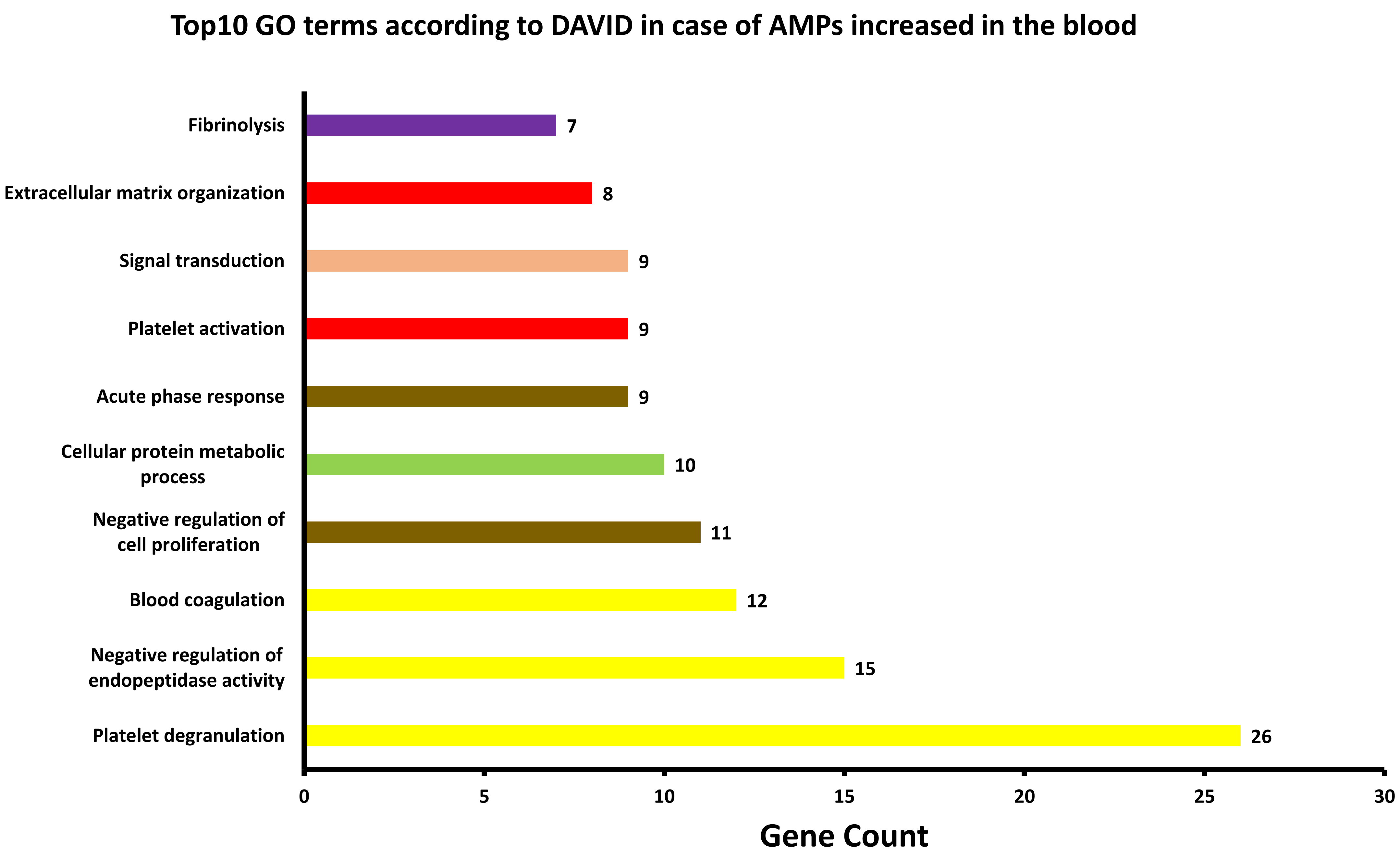
Top10 GO terms according to DAVID in case of AMPs increased in the blood
Fibrinolysis
7
Extracellular matrix organization
8
Signal transduction
9
Platelet activation
9
Acute phase response
9
Cellular protein metabolic process
10
Negative regulation of cell proliferation
11
Blood coagulation
12
Negative regulation of endopeptidase activity
15
Platelet degranulation
26
0
5
10
15
20
25
30
Gene Count

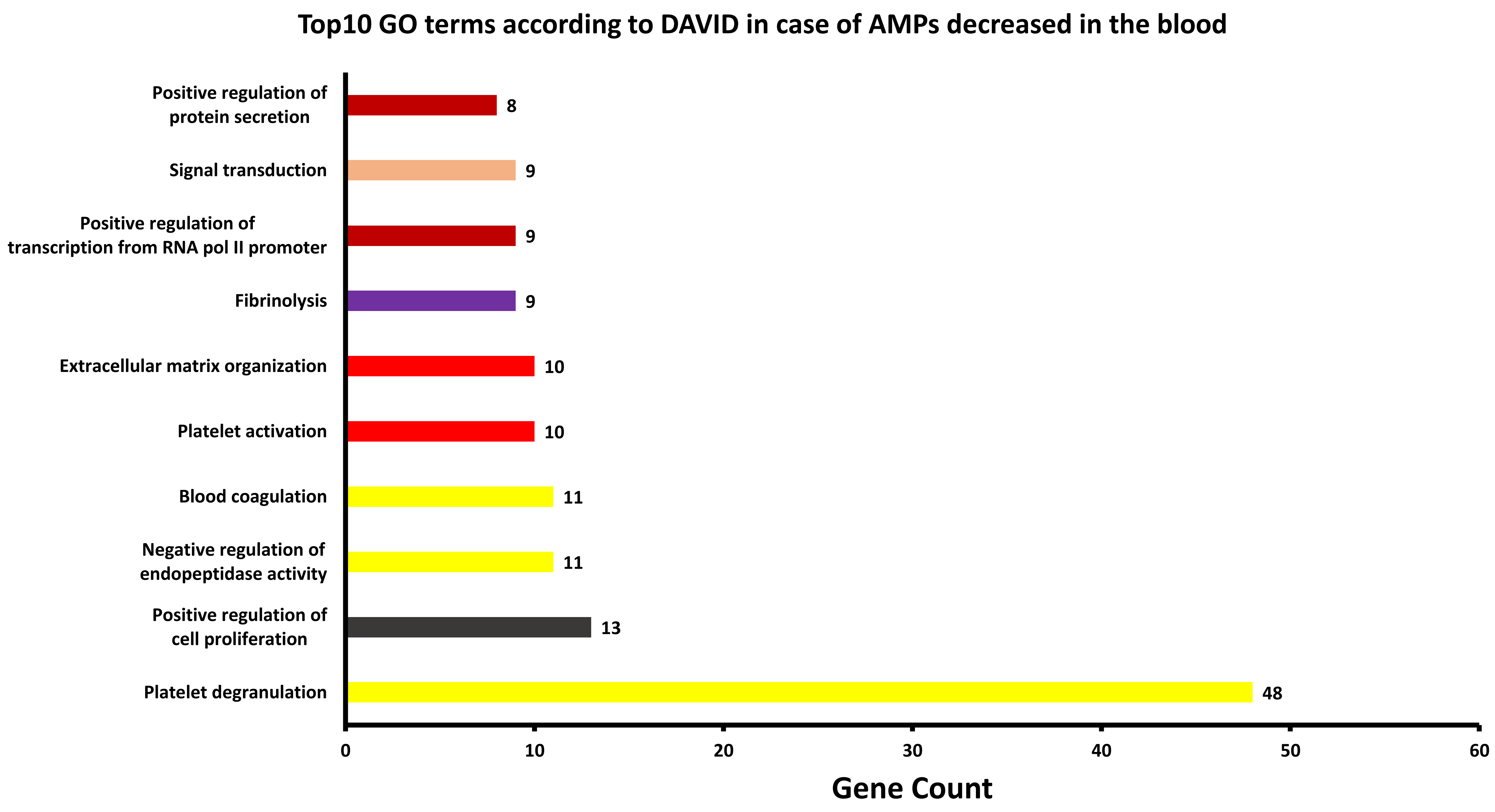
Top10 GO terms according to DAVID in case of AMPs decreased in the blood
Positive regulation of protein secretion
8
Signal transduction
9
Positive regulation of transcription from RNA pol II promoter
9
Fibrinolysis
9
Extracellular matrix organization
10
Platelet activation
10
Blood coagulation
11
Negative regulation of endopeptidase activity
11
Positive regulation of cell proliferation
13
Platelet degranulation
48
0
10
20
30
40
50
60
Gene Count

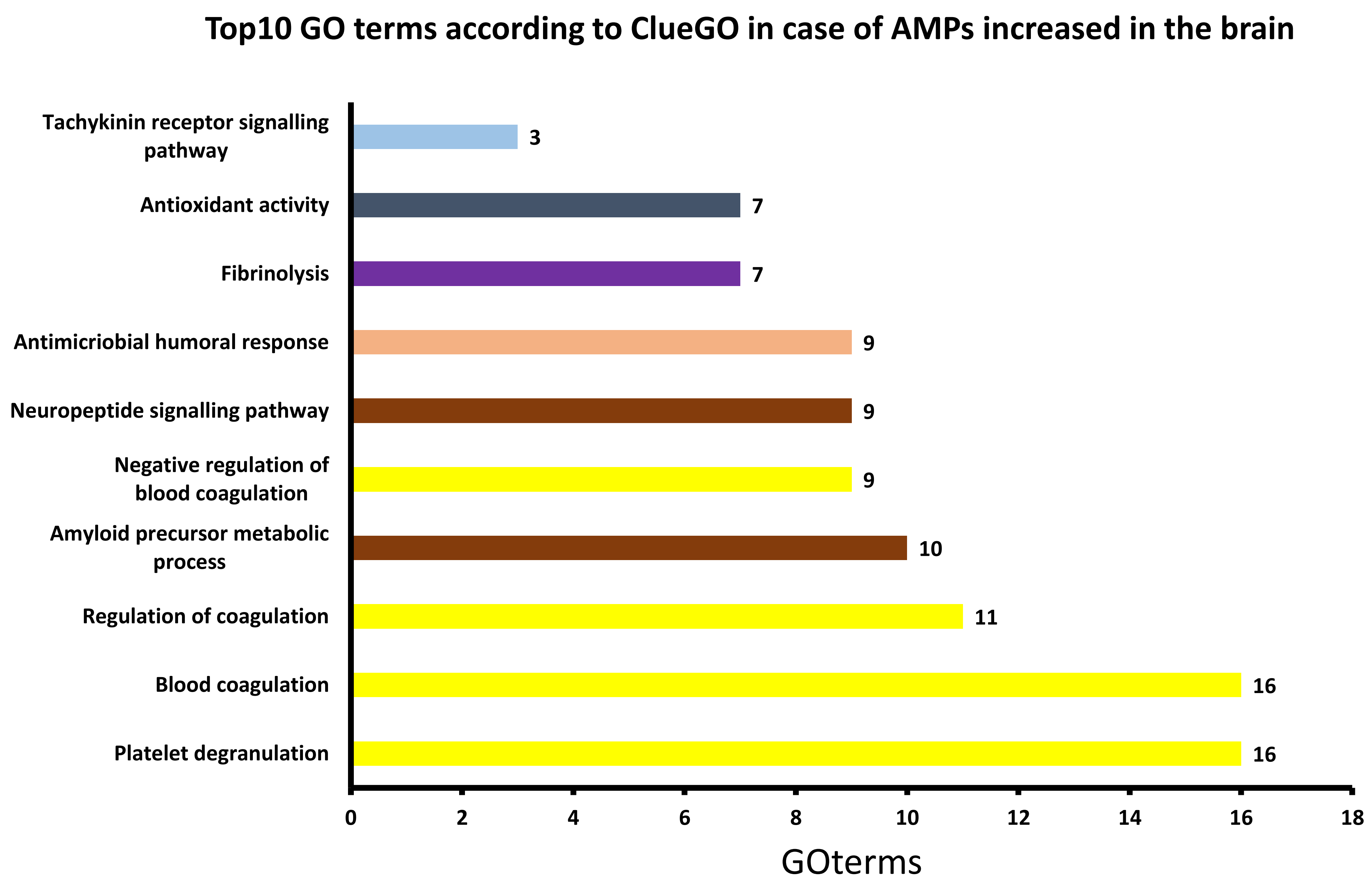
Top10 GO terms according to ClueGO in case of AMPs increased in the brain
Tachykinin receptor signalling pathway
3
Antioxidant activity
7
Fibrinolysis
7
Antimicriobial humoral response
9
Neuropeptide signalling pathway
9
Negative regulation of blood coagulation
9
Amyloid precursor metabolic process
10
Regulation of coagulation
11
Blood coagulation
16
Platelet degranulation
16
0
2
4
6
8
10
12
14
16
18
GOterms

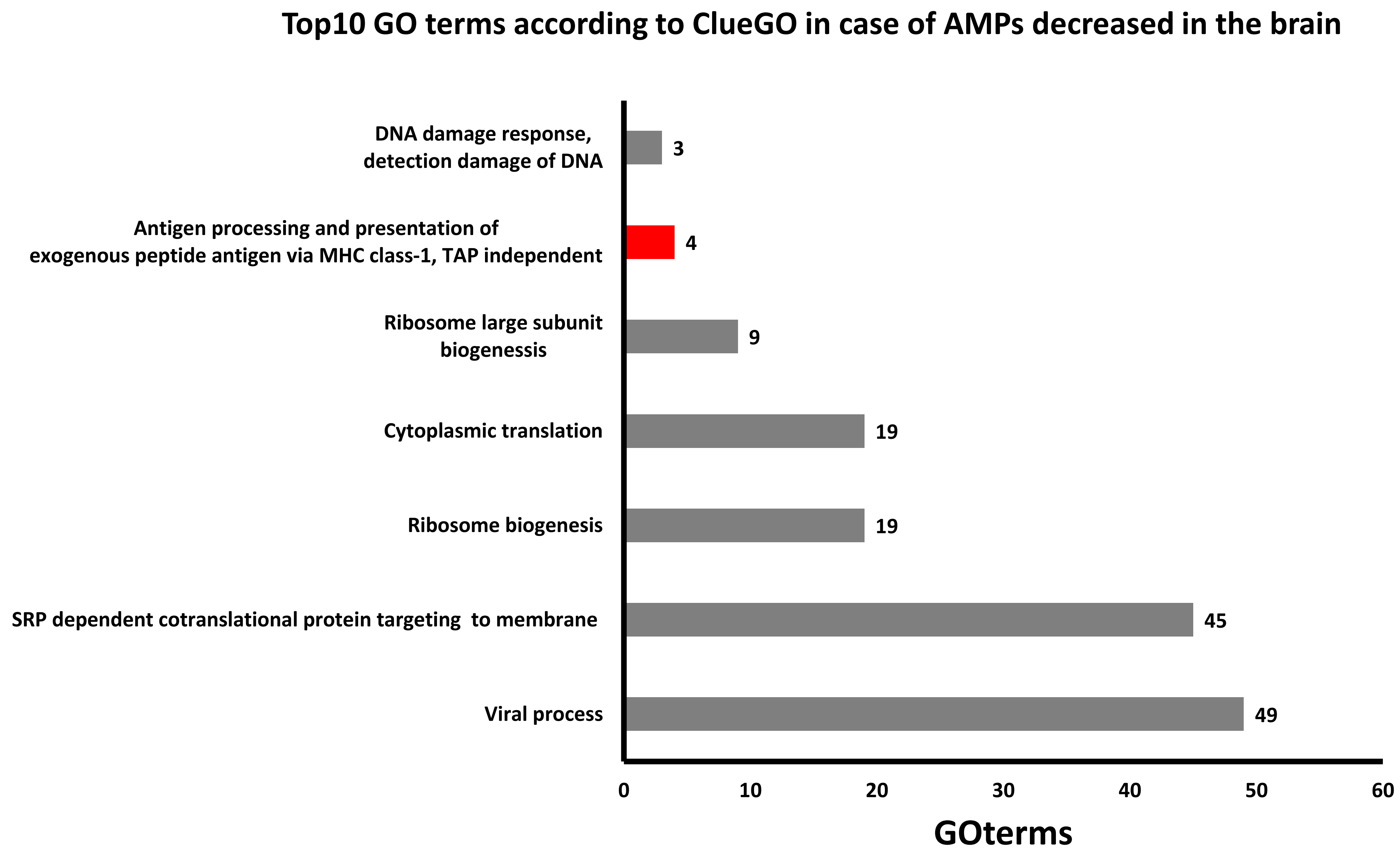
Top10 GO terms according to ClueGO in case of AMPs decreased in the brain
DNA damage response, detection damage of DNA
3
Antigen processing and presentation of exogenous peptide antigen via MHC class-1, TAP independent
4
Ribosome large subunit biogenessis
9
Cytoplasmic translation
19
Ribosome biogenesis
19
SRP dependent cotranslational protein targeting to membrane
45
Viral process
49
0
10
20
30
40
50
60
GOterms

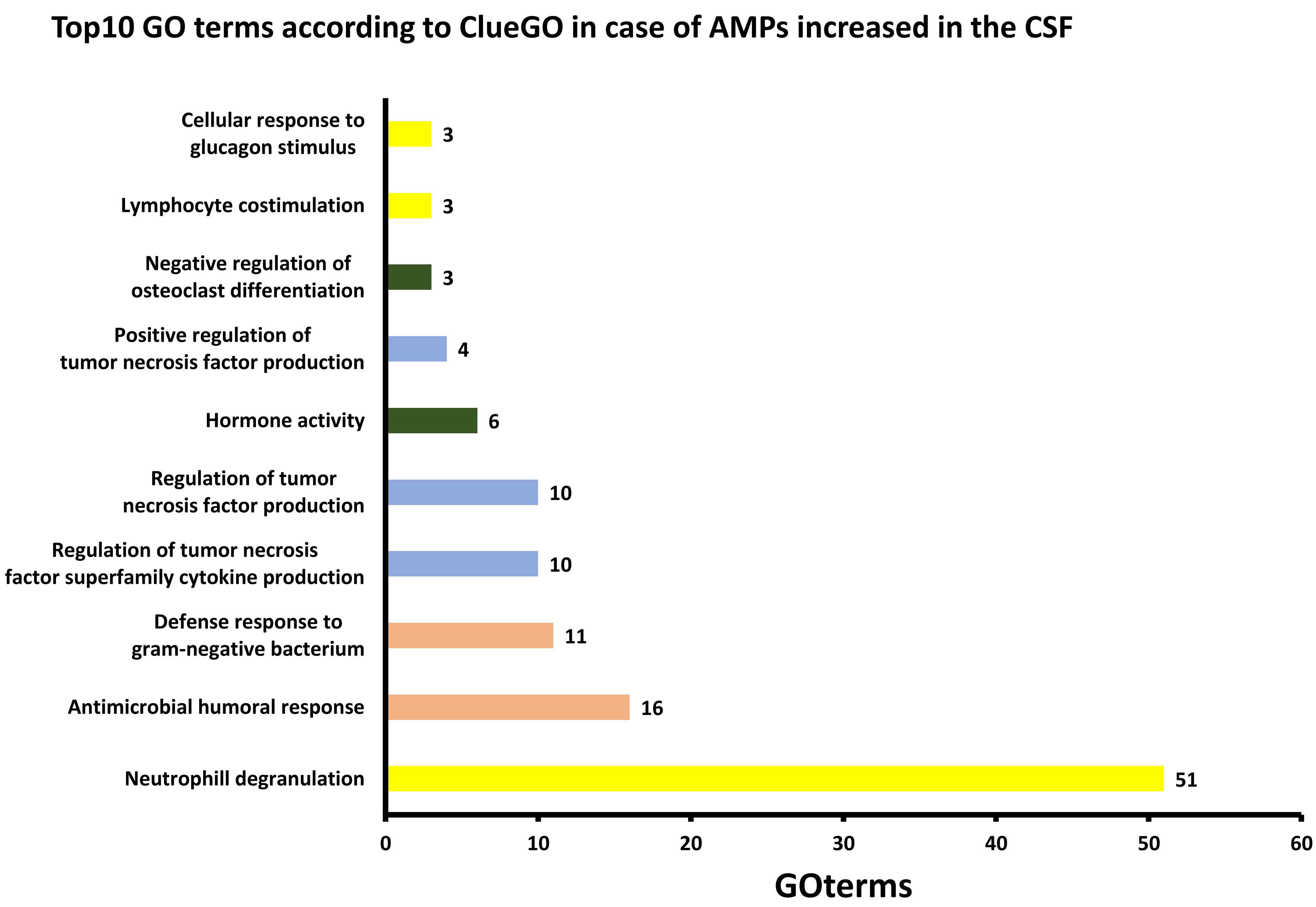
Top10 GO terms according to ClueGO in case of AMPs increased in the CSF
Cellular response to glucagon stimulus
3
Lymphocyte costimulation
3
Negative regulation of osteoclast differentiation
3
Positive regulation of tumor necrosis factor production
4
Hormone activity
6
Regulation of tumor necrosis factor production
10
Regulation of tumor necrosis factor superfamily cytokine production
10
Defense response to gram-negative bacterium
11
Antimicrobial humoral response
16
Neutrophill degranulation
51
0
10
20
30
40
50
60
GOterms

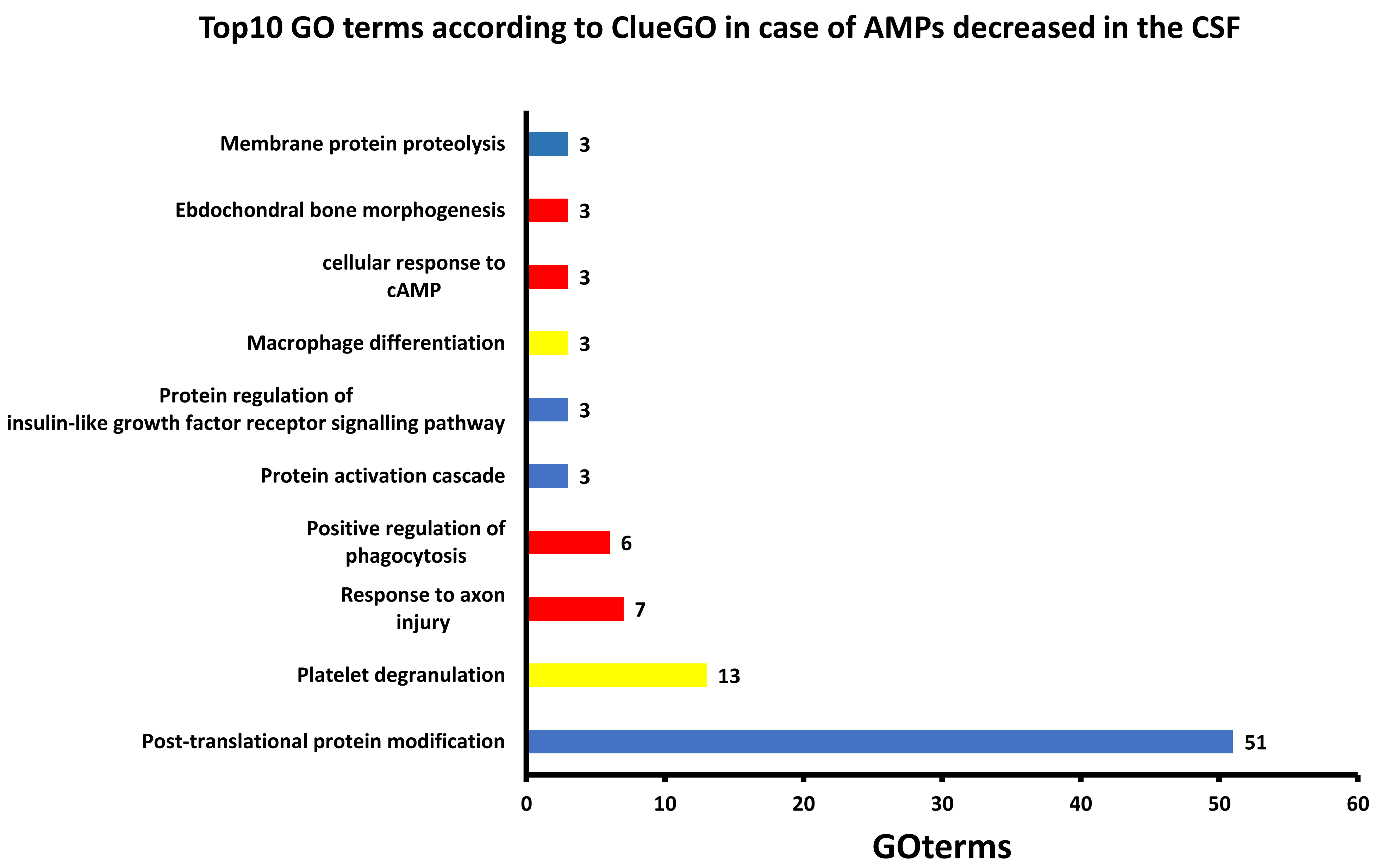

Top10 GO terms according to ClueGO in case of AMPs decreased in the CSF
Membrane protein proteolysis
3
Ebdochondral bone morphogenesis
3
cellular response to cAMP
3
Macrophage differentiation
3
Protein regulation of insulin-like growth factor receptor signalling pathway
3
Protein activation cascade
3
Positive regulation of phagocytosis
6
Response to axon injury
7
Platelet degranulation
13
Post-translational protein modification
51
0
10
20
30
40
50
60
GOterms

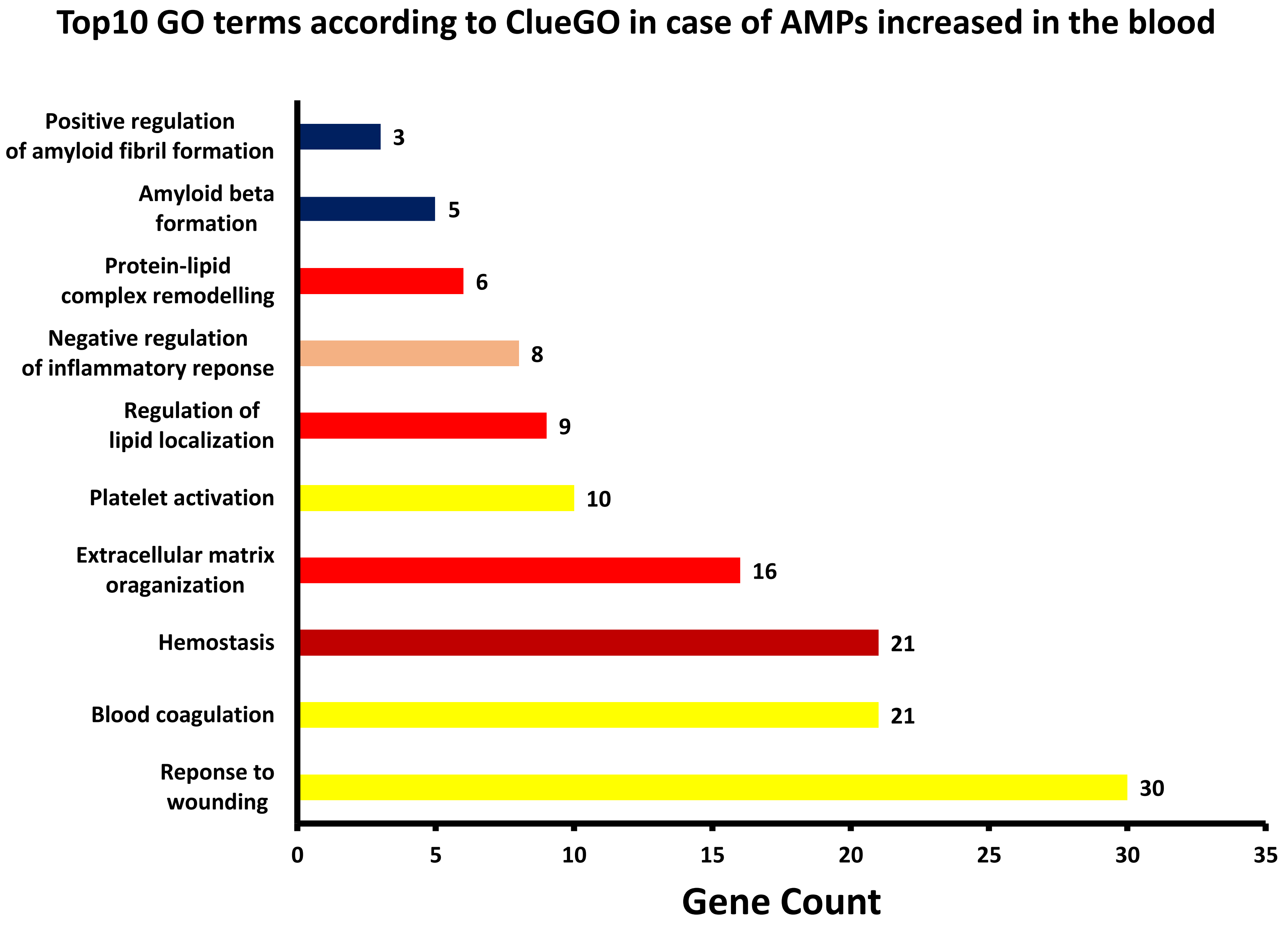

Top10 GO terms according to ClueGO in case of AMPs increased in the blood
Positive regulation of amyloid fibril formation
3
Amyloid beta formation
5
Protein-lipid complex remodelling
6
Negative regulation of inflammatory reponse
8
Regulation of lipid localization
9
Platelet activation
10
Extracellular matrix oraganization
16
Hemostasis
21
Blood coagulation
21
Reponse to wounding
30
0
5
10
15
20
25
30
35
Gene Count

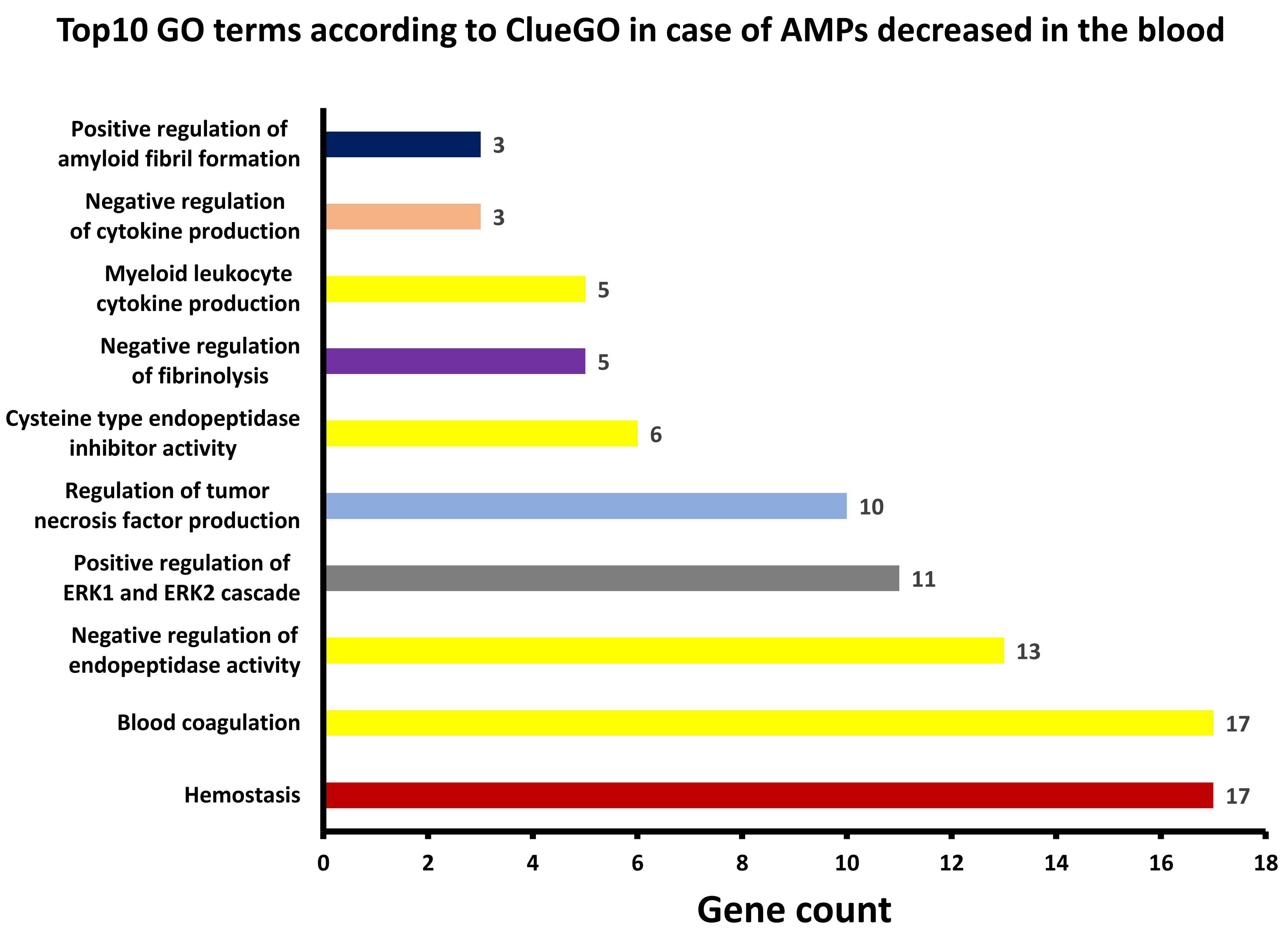
Top10 GO terms according to ClueGO in case of AMPs decreased in the blood
Positive regulation of amyloid fibril formation
3
Negative regulation of cytokine production
3
Myeloid leukocyte cytokine production
5
Negative regulation of fibrinolysis
5
Cysteine type endopeptidase inhibitor activity
6
Regulation of tumor necrosis factor production
10
Positive regulation of ERK1 and ERK2 cascade
11
Negative regulation of endopeptidase activity
13
Blood coagulation
17
Hemostasis
17
0
2
4
6
8
10
12
14
16
18
Gene count

Figure S4: Protein-protein interaction network of AMPs increased in brain in AD.

The AMPs showing a statistically significant increase in AD and their 50 first shell of interactors are shown. The rectangles indicate the proteins and the lines indicate the interaction between them. The proteins are labelled with their gene name

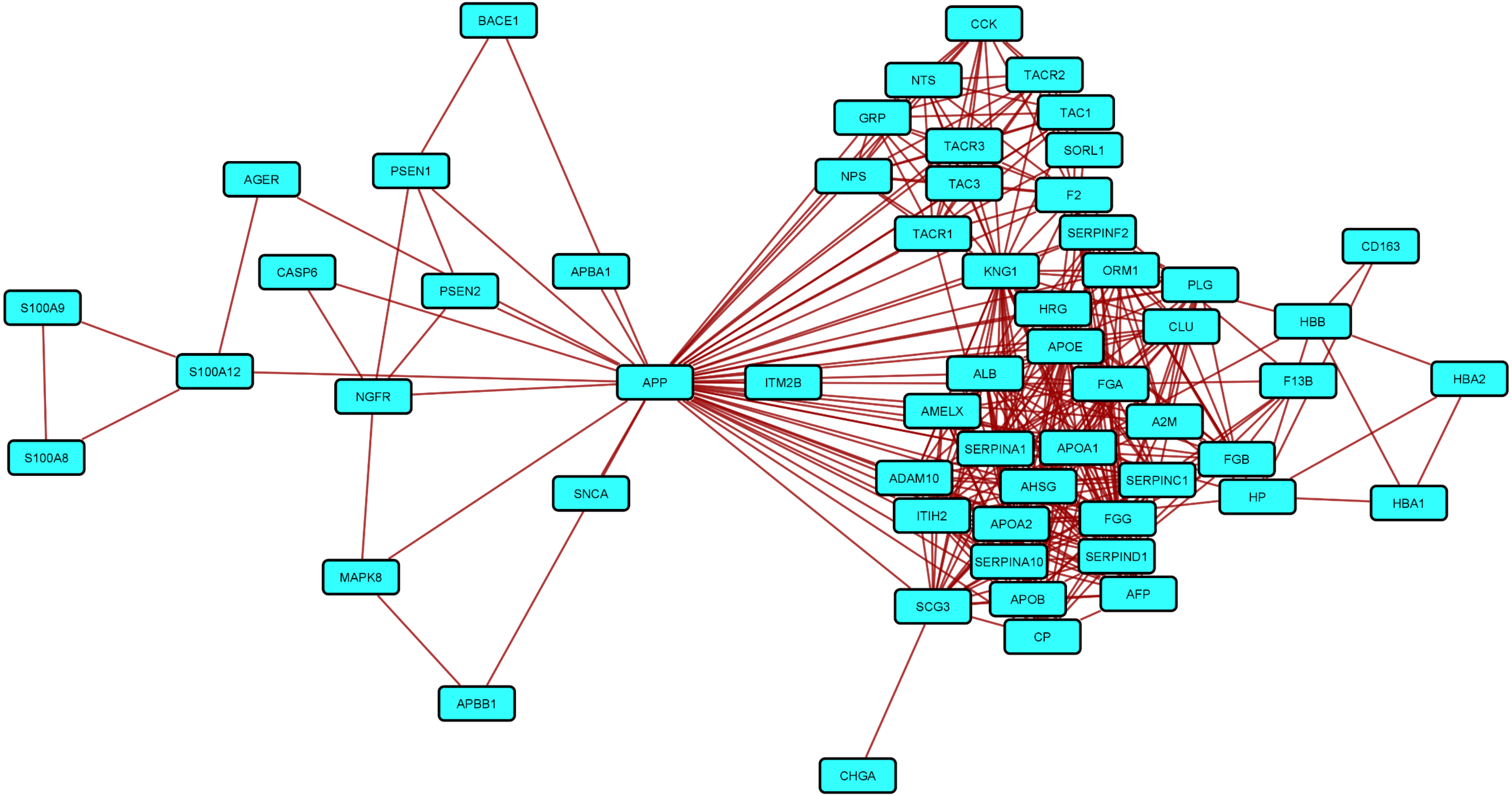
BACE1
CCK
NTS
TACR2
GRP
TAC1
TACR3
SORL1
AGER
PSEN1
NPS
TAC3
F2
TACR1
SERPINF2
CD163
APBA1
KNG1
ORM1
CASP6
PSEN2
PLG
S100A9
HRG
CLU
HBB
APOE
S100A12
ALB
F13B
HBA2
APP
ITM2B
FGA
NGFR
AMELX
A2M
SERPINA1
APOA1
FGB
ADAM10
SERPINC1
SNCA
AHSG
HP
HBA1
ITIH2
APOA2
FGG
S100A8
MAPK8
SERPINA10
SERPIND1
AFP
APOB
SCG3
CP
APBB1
CHGA

Figure S5: Gene interaction networks (GIN) for AMPs characteristic to AD A. GIN for AMPs increased in brain. **B.** GIN for AMPs decreased in brain. **C.** GIN for AMPs increased in CSF. **D.** GIN for AMPs decreased in CSF. **E.** GIN for AMPs increased in blood. **F.** GIN for AMPs decreased in blood. The circles represent a gene/protein and the lines indicate interactions. The lines with arrow represent activation, blocking lines represent inhibition, simple lines represent protein-protein interaction. Line color indicates the type of interaction: green color refers to activation, red color to inhibition, blue color binding, whereas yellow color shows co-expression. The red colour of protein label indicates AMPs whose amount changed in a statistically significant manner between AD and control samples, the blue colour shows their interacting partners with no AMP activity, while the green colour indicates interactors with AMP activity. On all panels the proteins are labelled with their gene name.

A

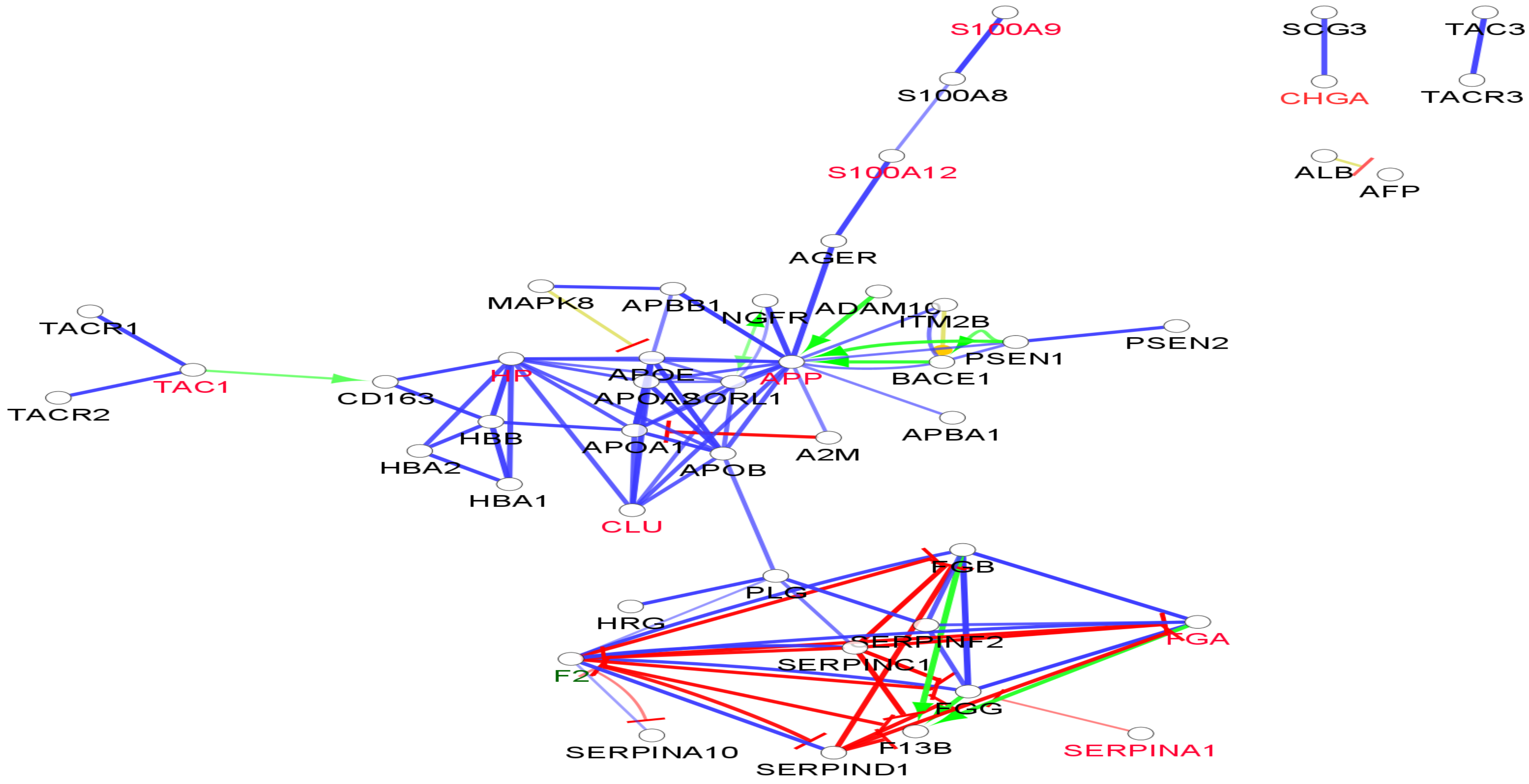

S100A9
SCG3
TAC3
S100A8
CHGA
TACR3
S100A12
ALB
AFP
AGER
MAPK8
APBB1
NGFR
ADAM10
ITM2B
TACR1
PSEN1
PSEN2
HP
APOE
APP
BACE1
TAC1
CD163
APOA2
SORL1
TACR2
APBA1
HBB
APOA1
A2M
HBA2
APOB
HBA1
CLU
FGB
PLG
HRG
SERPINF2
FGA
SERPINC1
F2
FGG
F13B
SERPINA10
SERPINA1
SERPIND1

B

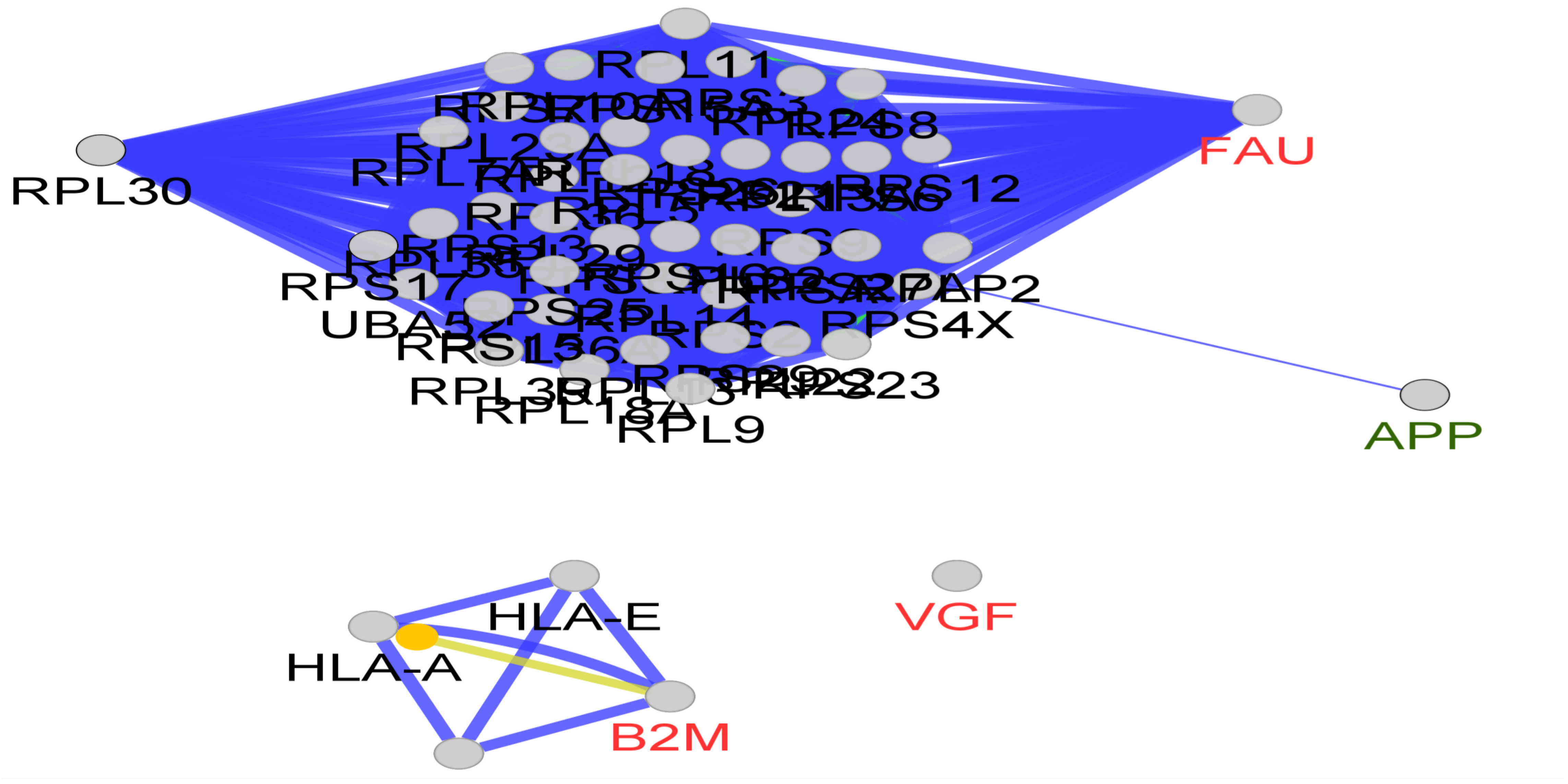

C

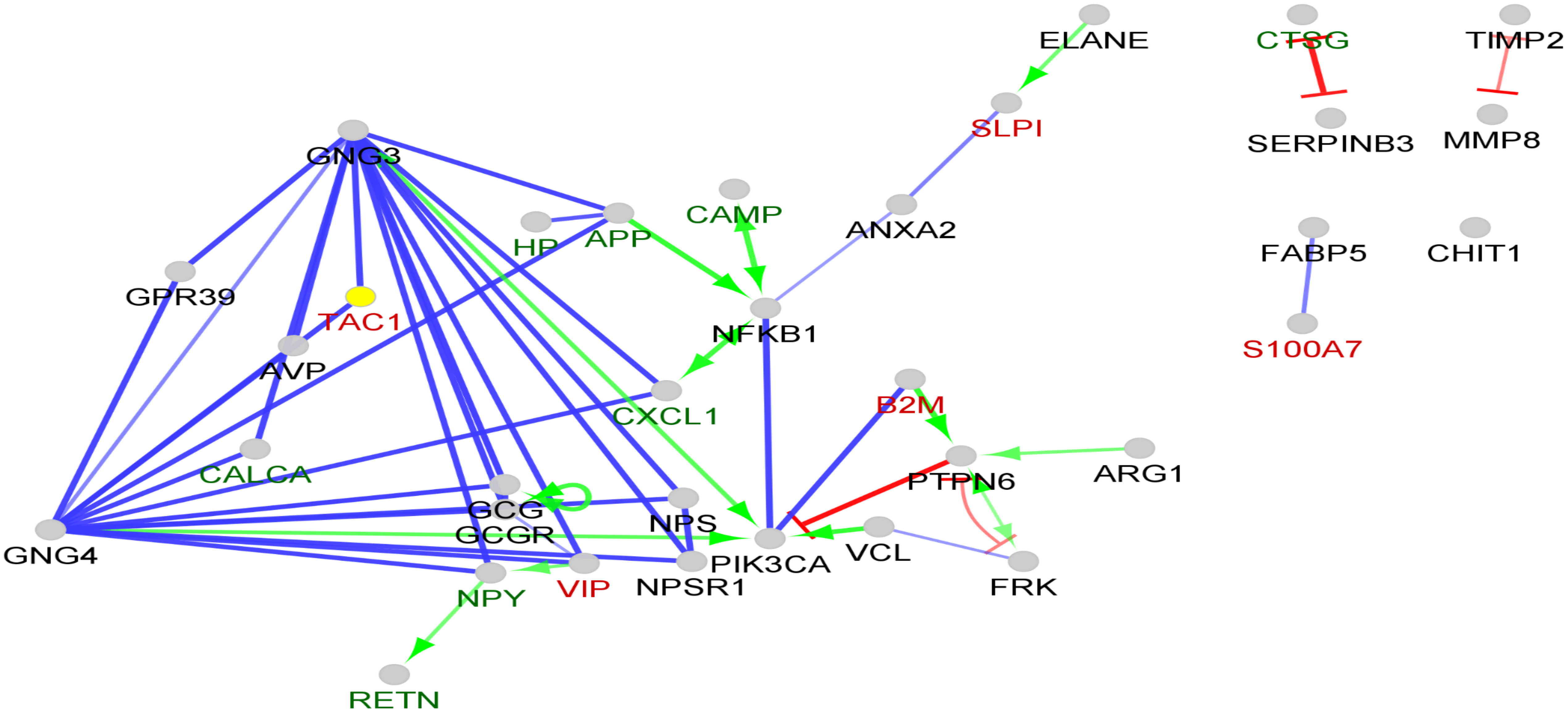

ELANE
CTSG
TIMP2
SLPI
SERPINB3
MMP8
GNG3
CAMP
ANXA2
HP
APP
FABP5
CHIT1
GPR39
TAC1
NFKB1
S100A7
AVP
CXCL1
B2M
CALCA
PTPN6
ARG1
GCG
NPS
GCGR
GNG4
PIK3CA
VCL
FRK
VIP
NPSR1
NPY
RETN

D

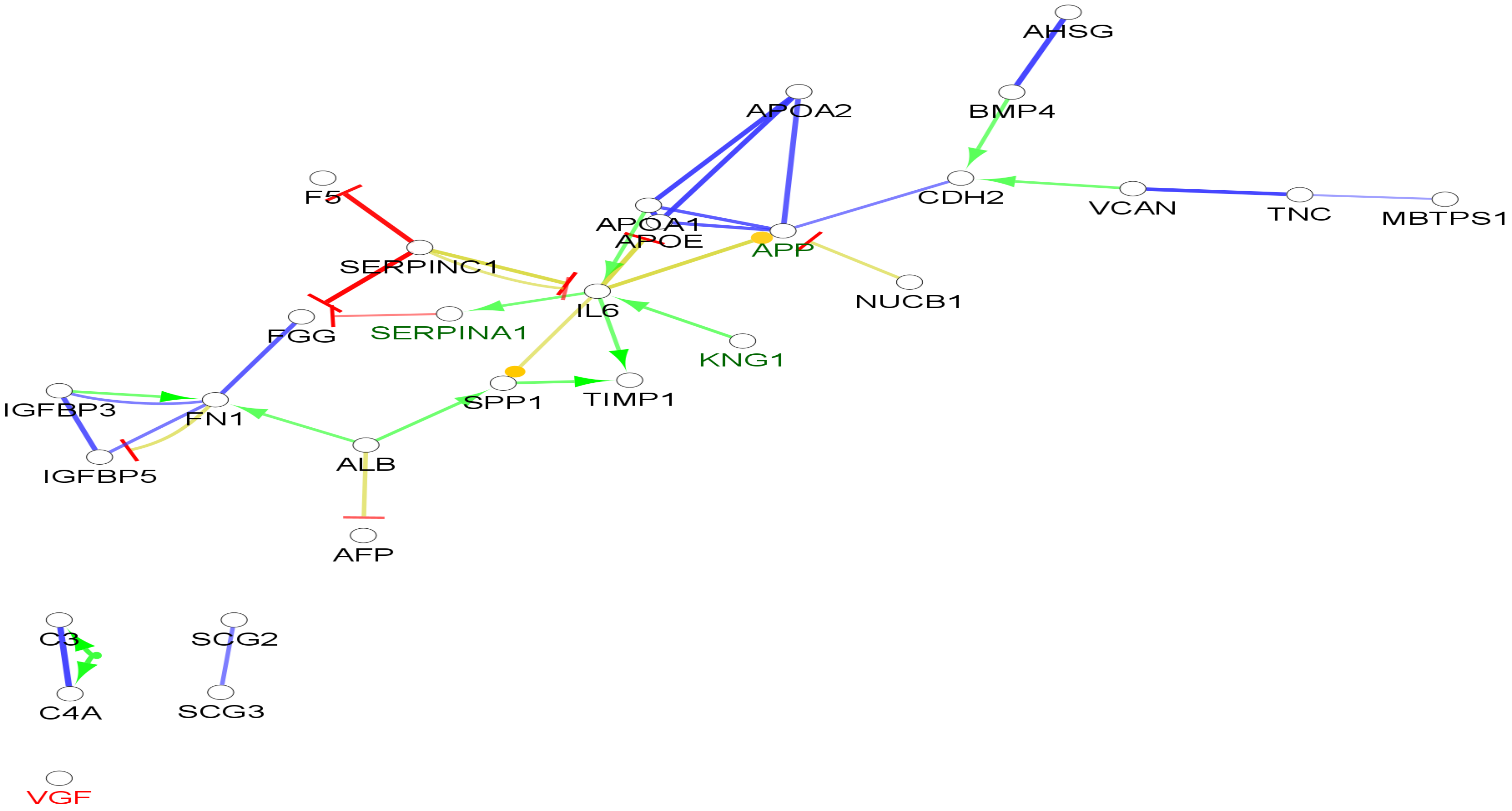
AHSG
BMP4
APOA2
F5
CDH2
VCAN
TNC
MBTPS1
APOA1
APOE
APP
SERPINC1
IL6
NUCB1
FGG
SERPINA1
KNG1
SPP1
TIMP1
IGFBP3
FN1
ALB
IGFBP5
AFP
C3
C4A
SCG2
SCG3
VGF

E

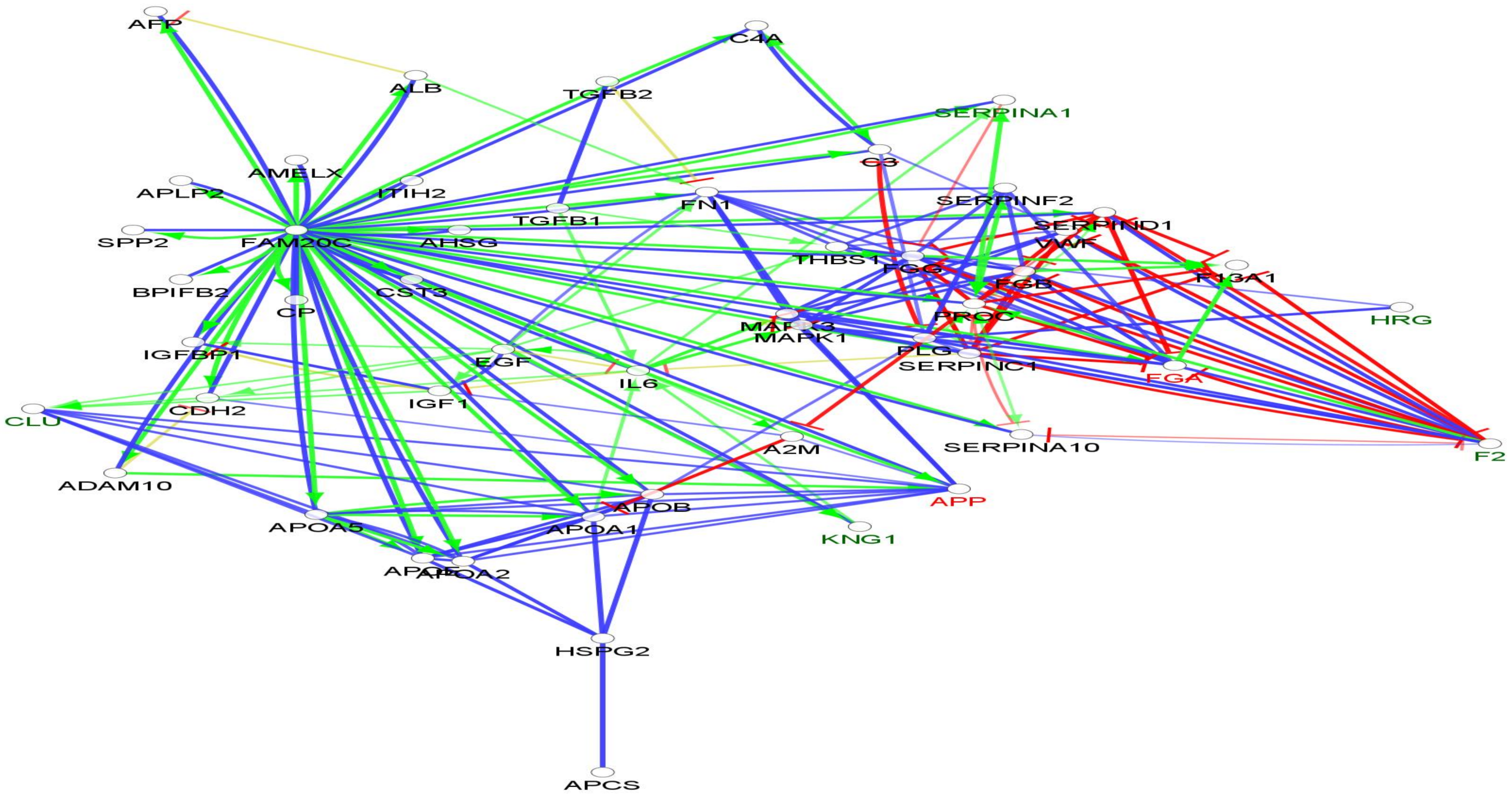

AFP
ALB
C4A
TGFB2
SERPINA1
C3
AMELX
APLP2
ITIH2
FN1
SERPINF2
TGFB1
SERPIND1
SPP2
FAM20C
AHSG
VWF
THBS1
FGG
F13A1
FGB
BPIFB2
CST3
CP
PROC
HRG
MAPK3
MAPK1
PLG
IGFBP1
EGF
SERPINC1
FGA
IL6
CDH2
IGF1
CLU
A2M
SERPINA10
F2
ADAM10
APP
APOB
APOA5
APOA1
KNG1
APOE
APOA2
HSPG2
APCS

F

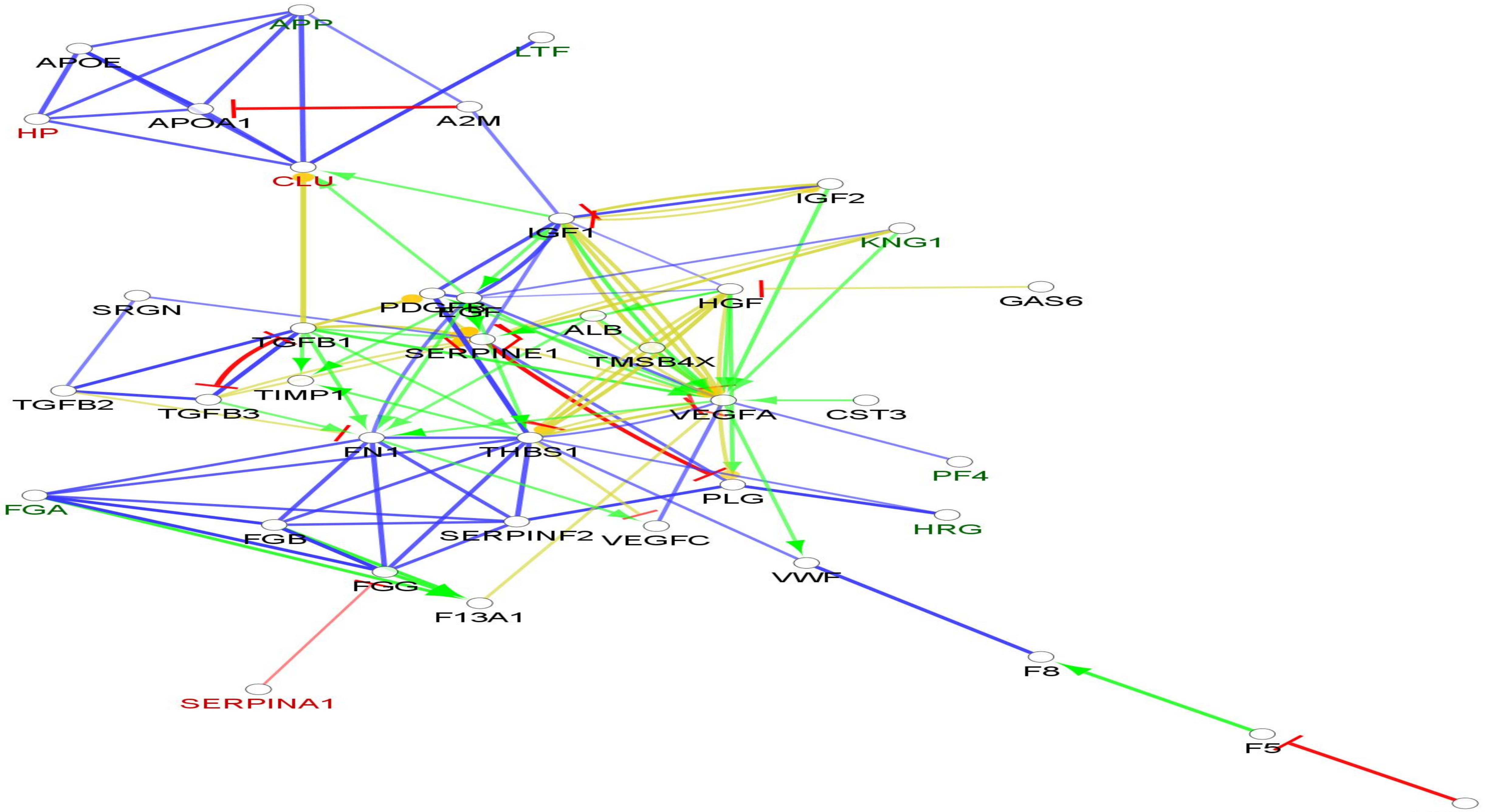
APP
LTF
APOE
APOA1
A2M
HP
CLU
IGF2
IGF1
KNG1
GAS6
SRGN
PDGFB
EGF
HGF
ALB
TGFB1
SERPINE1
TMSB4X
TIMP1
TGFB2
TGFB3
VEGFA
CST3
FN1
THBS1
PF4
FGA
PLG
HRG
FGB
SERPINF2
VEGFC
VWF
FGG
F13A1
F8
SERPINA1
F5
